# Gut Dysbiosis Drives Inflammatory Bowel Disease Through the CCL4L2‐VSIR Axis in Glycogen Storage Disease

**DOI:** 10.1002/advs.202309471

**Published:** 2024-06-18

**Authors:** Jiaoli Lan, Yuxin Zhang, Cuiyuan Jin, Huan Chen, Zexiong Su, Jiaxing Wu, Ni Ma, Xiaoyan Zhang, Yiyun Lu, Yongxin Chen, Xiaolu Zeng, Huiqiong Zhang, Guilang Zheng, Yueyu Sun, Chun Wang, Yan Hu, Yifei Wang, Yumei Liu, Zhaoyang Zeng, Liyun Shi, Jun He, Aihua Cao, Yichao Wang, Xu Pan, Gulei Jin, Ying Wang, Xun Jiang, Huiqing Shen, Qing Tang, Xiaoli Xie, Yuan Xiao, Xuemei Zhong, Xuchao Zhang, Liang Zeng, Liping Ye, Jing Xie, Lanlan Geng, Zhiling Li, Xiaohui Wu, Ying Wang, Ren Mao, Shaojun Zhang, Siyuan Huang, Suling Liu, Hanshi Zeng, Wanfu Xu, Sitang Gong, Yuxiong Guo, Min Yang

**Affiliations:** ^1^ Department of Pediatrics Guangdong Provincial People's Hospital Guangdong Academy of Medical Sciences Southern Medical University Guangzhou 510080 China; ^2^ Research Center of Medical Sciences Guangdong Provincial People's Hospital Guangdong Academy of Medical Sciences Guangzhou 510080 China; ^3^ Nanfang Hospital Southern Medical University Guangzhou 510515 China; ^4^ Institute of Translational Medicine Zhejiang Shuren University Hangzhou 310015 China; ^5^ Department of Gastroenterology Guangzhou Women and Children's Medical Center Guangzhou Medical University Guangzhou 510623 China; ^6^ Hunan Provincial Key Laboratory of Regional Hereditary Birth Defects Prevention and Control Changsha Hospital for Maternal & Child Health Care Affiliated to Hunan Normal University Changsha 410007 China; ^7^ Department of Pediatrics Shandong University Qilu Hospital Jinan Shandong 250063 China; ^8^ National Health Commission (NHC) Key Laboratory of Birth Defect for Research and Prevention Hunan Provincial Maternal and Child Health Care Hospital Changsha Hunan 410008 China; ^9^ Department of Basic Medical Sciences School of Medicine Tsinghua University Beijing 100084 China; ^10^ Institute of Bioinformatics College of Agronomy and Biotechnology Zhejiang University Hangzhou Zhejiang 277599 China; ^11^ Division of Pediatric Gastroenterology and Nutrition Xinhua Hospital Affiliated to Shanghai Jiao Tong University School of Medicine Shanghai 200092 China; ^12^ Department of Pediatrics The Second Affiliated Hospital Air Force Military Medical University Xi'an 710032 China; ^13^ Department of gastroenterology Beijing Children's Hospital Capital Medical University National Center for Children's Health Beijing 100045 China; ^14^ Department of Pediatrics The First Affiliated hospital of Guangxi Medical University Nanning 530021 China; ^15^ Chengdu Women's and Children's Central Hospital School of Medicine University of Electronic Science and Technology of China Chengdu 610073 China; ^16^ Department of Pediatrics Ruijin Hospital Shanghai Jiao Tong University School of Medicine Shanghai 200025 China; ^17^ Department of gastroenterology Capital Institute of Pediatrics No. 2 Yabao Road Beijing 100020 China; ^18^ Guangdong Lung Cancer Institute Medical Research Center Guangdong Provincial People's Hospital Guangdong Academy of Medical Sciences Guangzhou 510080 China; ^19^ Guangdong Provincial Key Laboratory of Translational Medicine in Lung Cancer Guangdong Provincial People's Hospital Guangdong Academy of Medical Sciences Guangzhou 510080 China; ^20^ Department of pathology Guangzhou Women and Children's Medical Center Guangzhou Medical University Guangzhou 510623 China; ^21^ Department of Gastroenterology The First Affiliated Hospital Sun Yat‐Sen University Guangzhou 510062 China; ^22^ Academy for Advanced Interdisciplinary Studies Peking University Beijing 100091 China; ^23^ Clinical Laboratory Guangdong Provincial People's Hospital Guangdong Academy of Medical Sciences Guangzhou 510080 China

**Keywords:** CCL4L2‐VSIR, glycogen storage disease, gut microbiota, inflammatory bowel disease, macrophage

## Abstract

Patients with glycogen storage disease type Ib (GSD‐Ib) frequently have inflammatory bowel disease (IBD). however, the underlying etiology remains unclear. Herein, this study finds that digestive symptoms are commonly observed in patients with GSD‐Ib, presenting as single or multiple scattered deep round ulcers, inflammatory pseudo‐polyps, obstructions, and strictures, which differ substantially from those in typical IBD. Distinct microbiota profiling and single‐cell clustering of colonic mucosae in patients with GSD are conducted. Heterogeneous oral pathogenic enteric outgrowth induced by GSD is a potent inducer of gut microbiota immaturity and colonic macrophage accumulation. Specifically, a unique population of macrophages with high CCL4L2 expression is identified in response to pathogenic bacteria in the intestine. Hyper‐activation of the CCL4L2‐VSIR axis leads to increased expression of AGR2 and ZG16 in epithelial cells, which mediates the unique progression of IBD in GSD‐Ib. Collectively, the microbiota‐driven pathomechanism of IBD is demonstrated in GSD‐Ib and revealed the active role of the CCL4L2‐VSIR axis in the interaction between the microbiota and colonic mucosal immunity. Thus, targeting gut dysbiosis and/or the CCL4L2‐VISR axis may represent a potential therapy for GSD‐associated IBD.

## Graphical abstract

Fairly large and strict‐controlled gut microbiota cohort shows that dysbiosis in the gut is a significant mediator to the GSD‐associated comorbidities such as inflammatory bowel disease. Single cell sequencing combined in‐vivo and ‐vitro experiments reveal the gut dysbiosis induces high expression of CCL4L2‐VSIR axis in GSD‐Ib, which plays active role in the interaction between microbiota and colonic mucosal immunity.

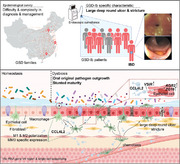

## 1 Introduction

Glycogen storage disease (GSD) is a rare, inherited metabolic disorder that causes improper glycogen storage in the body, primarily in the liver and/or muscles.^[^ ^1^ ^]^ The overall incidence of GSD is estimated to be 1 in 20 000–43 000 live births.^[^ ^2^ ^]^ Mutations in the gene encoding glucose‐6‐phosphatase (*G6PC*) cause GSD type Ia (GSD‐Ia), while mutations in glucose‐6‐phosphate translocase (*G6PT/SLC37A4*) cause GSD type Ib (GSD‐Ib). Patients with GSD‐Ib present with severe hypoglycemia and digestive symptoms.^[^ ^1^, ^2^ ^]^ Notably, > 70% of patients with GSD‐Ib have endoscopically confirmed inflammatory bowel disease (IBD),^[^ ^3^ ^]^ while IBD are occasionally associated with GSD‐Ia.^[^ ^4^ ^]^ A previous study attributed IBD to neutropenia and neutrophil dysfunction in GSD‐Ib,^[^ ^3^ ^]^ while GSD‐Ia was not associated with neutrophil defects. In addition, an overload of uncooked cornstarch (UCCS) can lead to a low fecal pH by favoring microbes capable of utilizing complex carbohydrates to the detriment of others, which causes gut dysbiosis and contributes to the development of IBD in GSD‐I.^[^ ^5^ ^]^ However, such conditions have not been reported for other types of GSD that also use UCCS. Studying the gastrointestinal symptoms of various types of GSD is beneficial not only for analyzing the effects of UCCS on the intestinal microenvironment, but also for enhancing our understanding of the development of GSD‐ associated IBD.

Gut microorganisms and impaired host immune responses play key roles in IBD progress.^[^ ^6^, ^7^, ^8^, ^9^, ^10^ ^]^ Despite the limited cohort size and genetic type, previous studies have indicated dramatic alterations in the microbiota and short‐chain fatty acids in the gut of patients with GSD,^[^ ^11^, ^12^, ^13^ ^]^ highlighting the pressing need for comprehensive observation. Various innate immune cells maintain the gut homeostasis. Macrophages are immune cells found in close proximity to the epithelial cells in the colon. Recent studies have suggested a causal role for altered monocyte‐macrophage transition in intestinal inflammation in patients with IBD.^[^ ^14^ ^]^ In addition, activated macrophages largely contribute to the release of cytokines and chemokines into the gut. Dysregulated cytokines and chemokines are implicated in the development and progression of IBD.^[^ ^15^ ^]^ However, a deeper understanding of the interactions between gut microorganisms and immune cells is lacking, and the key cytokines and chemokines contributing to GSD remain unclear.

Herein, we describe the gastrointestinal symptoms in addition to the disease‐specific clinical features of GSD. We revealed the endoscopic characteristics of deep round ulcers and structures in GSD‐Ib, which differed from the typical IBD features, including linear ulceration or cobblestone mucosa. We found that enteric extension of oral pathogens induced microbiota immaturity and gut dysbiosis, which led to the accumulation of a unique high CCL4L2 expressed group of macrophages in GSD. The interaction between macrophages and epithelial cells through the CCL4L2‐VSIR axis promotes the expression of mucin‐related genes, such as AGR2, thereby regulating mucosal barrier function.

## 2 Results

### 2.1 Epidemiological Surveys Reveal the Complexity of Clinical Characteristics and Medical Management of Patients with GSD in China

**Figure 1:**
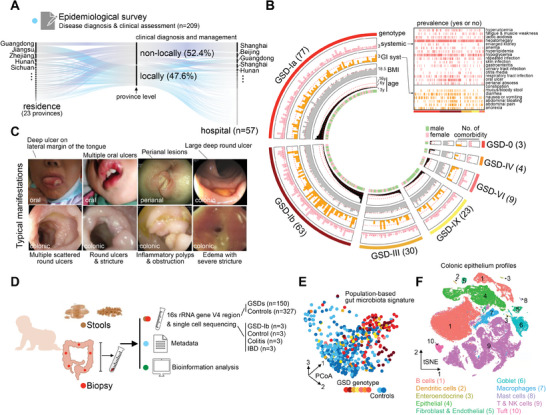
Main clinical manifestation of patients with GSD in China. A) Sankey diagram showing the current treatment‐seeking condition of families with GSD in China. B) Circos showing the summative comorbidity and demographic characteristics in patients with GSD from our epidemiological surveys (n=209). Heatmap (on the right part) showing the prevalence of each comorbidity in patients. Color block representing the patient suffers from the comorbidity (see Experimental Section and also Table S1, Supporting Information). C) Typical GI manifestations in patients with GSD‐Ib were included in the clinical observation cohort. D) Study design and sample collection for the present study. E) 3‐D Bray–Curtis dissimilarity based principal coordinate analysis (PCoA) displaying the distributions of patient at OTU level. F) tSNE plot displaying 89 735 cells from 12 children separated into ten major cell types. GSD, glycogen storage disease; GI, gastrointestinal; OTU, operational taxonomic unit.

To investigate the epidemiological and clinical characteristics of GSD in mainland China, we conducted two large multicenter studies and reported 209 GSD cases through questionnaires from October 2020 to June 2021 (Table S1, Supporting Information). Notably, 52.4% of patients clinical diagnoses and management were province‐level allochthonous, and the registered hospitals were mainly from relatively developed regions. (**Figure** **1**A). Six types of GSD, type 0 (*Gys2*), I (*G6PC* and *SLC37A4*), III (*AGL*), IV (*GBE1*), VI (*PYGL*), and IX (*PHKA*), were included, with the most common being type I (66.9%) (Figure 1B; Table S1, Supporting Information). The general clinical characteristics of GSD include hypoglycemia, hepatomegaly, acidosis (lactic acidosis), hyperlipidemia, hyperuricemia, fatigue, and muscle weakness (Figure 1B).^[^ ^16^ ^]^ Complaints, such as aphthous stomatitis, perianal lesions, gastroenteritis, respiratory tract infections, and skin infections, were more prevalent in GSD type I (Figure 1B; Table S1, Supporting Information). Anorexia, vomiting, diarrhea, mucus/bloody stools, abdominal pain, and abdominal distension were common gastrointestinal complications in patients with GSD, particularly in the GSD‐Ib group (77.8%) (Figure 1B).^[^ ^3^ ^]^ Although 94.3% of patients maintained their blood sugar levels using the UCCS, limited effects were observed in reducing the incidence of comorbidities in patients with GSD (Figure 1B).

In our multicenter endoscopic surveillance study, we recruited 32 patients with GSD. These patients exhibited active gastrointestinal symptoms during the endoscopic examination. Of the 32 patients, 27 had GSD‐Ib and 25 were initially diagnosed with IBD. We found that the colonoscopy features in patients with GSD‐Ib were single or scattered multiple large, deep, round ulcers and strictures (Figure 1C; Figure S1A, Supporting Information), which differs from the typical IBD features of linear ulceration or cobblestone mucosa (Figure S1B, Supporting Information). In particular, the initial colonoscopy showed colonic stenosis in ten patients (40%, 10/25) and colonic perforation in one patient. This finding reveals the complexity of gastrointestinal and systemic comorbidities in patients with GSD.

To reveal the potential role of the interaction between the gut microbiota and colonic mucosae in IBD in patients with GSD, we collected 477 stool samples, including 150 stool samples from patients with GSD and 327 from healthy controls (Figure 1D). Using *16S* rRNA gene sequencing, we identified distinct profiles of gut microbiota in patients with GSD (Figure 1E). In addition, we performed single‐cell RNA sequencing (scRNA‐seq) of the colonic mucosa from healthy controls (n = 3) and patients with colitis (n = 3), IBD (n = 3), and GSD‐Ib (n = 3) (Figure 1D). Ten groups of cells were identified using unsupervised clustering (Figure 1F).

### 2.2 GSD Genotypes Dominate Top Causes of Gut Microbiota Variation

**Figure 2:**
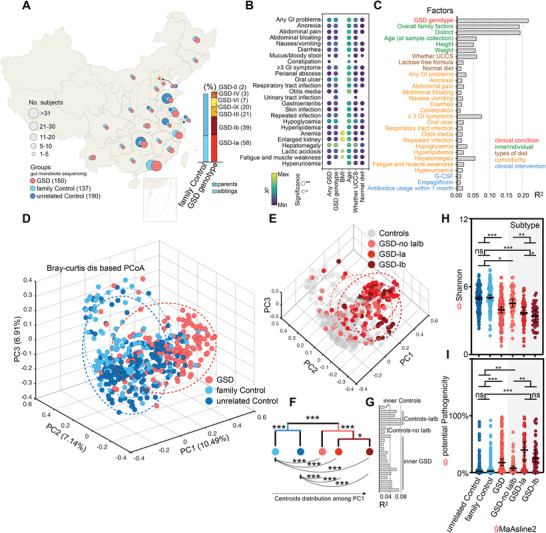
Clinical condition of GSD induces the most variations on the gut microbiota across multi‐factorial host effect. A) Summative overview of geographical and clinical characteristics of the present studied cohort. A total of 477 patients are recruited from 23 provinces in China and the histogram showing the parental relationship and genetic composition of the family Control and GSD groups. Basemap is from the Resource and Environment Science and Data Center (https://www.resdc.cn/Datalist1.aspx?FieldTyepID=20,0). B) Distributional correlation of individual factors is quantified by Mantel tests from metadata. FDR‐corrected statistical significance. C) Horizontal bars illustrating the amount of variance/effect size (R^2^) of each significant factor on gut microbiota variations. Factors are colored according to metadata categories. D,E) The PCoA space showing the gut microbial composition shift between groups. Plots representing the specific patients are colored according to their preliminary group definitions (D) or disease genotype (E). F,G) Differences (F) and the amount effect size (G) of beta‐diversity variance among different grouping methods are obtained by PERMANOVA respectively. H,I) Shannon index (H) and potential pathogenicity (I) among each group. Median with 95% confidence interval (CI) and paired‐wise Wilcoxon test with Bonferroni–Holm Correction. ġ (in red) indicating the microbial feature shows significant association with the clinical condition of GSD after adjusting for age, gender, district and BMI. ^*^ *p* < 0.05, ^**^ *p* < 0.01, ^***^ *p* < 0.001, and ^****^ *p* < 0.0001. FDR, false discovery rate; GI, gastrointestinal; GSD, glycogen storage disease.

The gut microbiota is substantially influenced by multiple factors, including diet, region, and medical conditions.^[^ ^17^ ^]^ To study the gut microbiota profiles of patients with GSD under multivariable conditions, 477 fecal samples were collected from 150 patients with GSD, 137 family controls, and 190 unrelated healthy controls (**Figure** **2**A; Table S2, Supporting Information). Disease conditions significantly correlated with the incidence of many comorbidities, particularly hepatomegaly, hypoglycemia, and gastrointestinal problems (Figure 2B; Figure S2A,B, Supporting Information).

Next, we evaluated the effect size (R^2^) of significant factors affecting the gut microbiota composition and found 45 accredited factors in our metadata (Table S3, Supporting Information). Notably, GSD genotypes predominantly affected the gut microbiota composition. Overall, family factors (family group paired comparison) and district of residence were the second and third most important factors affecting the gut microbiota composition (Figure 2C; Figure S2C, Supporting Information). Factors, such as diet are known to influence the gut microbiota; however, in our cohort, UCCS (94.7%), lactose‐free diet, or normal diet showed a limited impact on the gut microbiota compared to the GSD genotype (Figure 2C). Notably, the daily intake of UCCS did not affect the gut microbiota (Table S3, Supporting Information).

Although considerable inter‐individual variation existed in the same group, discrete distributions of gut microbiota between patients with GSD and their family or unrelated controls were observed (Figure 2D). To compare the gut microbiota of the GSD subgroups, we grouped the GSD genotypes with sample sizes below 20, GSD‐0, IV, and VI, as GSD‐other (Figure S2D, Supporting Information) and found no statistical differences between GSD‐III, IX, and the others (Figure S2E, Supporting Information). We then grouped the three groups of patients into GSD‐no Ia–Ib (Figure 2E; Figure S2D, Supporting Information) and found significant differences between the two control groups and the GSD‐Ia, Ib, and no‐IaIb groups (*P* < 0.0001, permutational multivariate analysis (PERMANOVA); Figure 2F). GSD‐Ia was also significantly different from GSD‐Ib (*P* = 0.031), and these two genotypes were associated with most gut microbiota variations in both intra‐ and inter‐subgroup analyses (Figure 2G; Figure S2D,F, Supporting Information).

### 2.3 Distinct Gut Pathogen Extension Disrupts Gut Microbiota Maturation in Patients with GSD

After adjusting for covariates, we found that a significantly decreased Shannon index and increased potential pathogenicity were representative characteristics of the gut microbiota in patients with GSD in terms of ecological levels (Figure 2H,I). At the community level, the percentage of facultative anaerobic bacteria significantly increased, and that of anaerobic bacteria decreased correspondingly (Figure S2G,H, Supporting Information).

**Figure 3:**
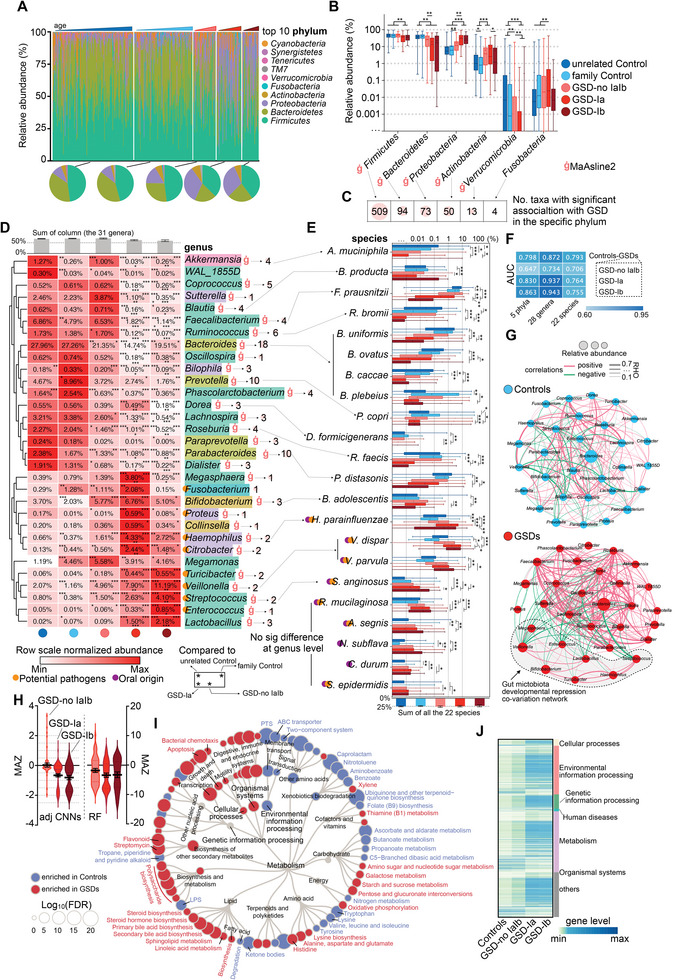
Distinct extension of gut pathogens stunted gut microbiota maturation in GSD patients. A) Relative abundance and the grouped‐average microbiota composition of the top 10 phyla in the 477 patients (the above part) and each group (the blow pie charts). B,C) Box‐whisker plots (B) illustrating the abundance shifts of gut microbiota at phylum level among groups. + indicating the mean values of abundance. The number of microbial taxa with significant association with GSD under multivariable condition of each phylum are shown in (C). D) Heatmap showing the mean abundance of the 31 significant disrupted genera between groups. Bar graph (on the top) indicating their accumulated abundance in each group (mean values ± SEM). E) Relative abundance of the 22 significant changed species among groups. The accumulated abundance of the 22 species in each group are shown in bar graph below. + indicating the mean values of abundance. All the selected species exhibited in E were also associated with GSD (ġ). Paired‐wise Wilcoxon test with Bonferroni–Holm correction. F) Heatmap showing the AUC values of the models built with the selected microbial taxa features (features with both significant abundance changes and GSD association) in each cohort. The values refer to the average value of the tenfold cross‐validation. G) Abundance correlations among the 31 genera in patients with GSD and controls. Only significant (FDR < 0.05) absolute correlations above 0.1 are shown. H) Violin plot showing the microbiota‐for‐age Z score (MAZ) of the patients inner GSD subgroups calculated by random forest regression (RF) and adjusted convolutional neural networks (CNNs). Mean values ± SEM. FDR, false discovery rate; GSD, glycogen storage disease. I,J) Functional hierarchical tree showing significantly altered gut microbial function in patients with GSD. Heatmap of KOs that differed significantly between groups and associated with GSD are shown in (J). ^*^ *p* < 0.05, ^**^ *p* < 0.01, and ^***^ *p* < 0.001. GSD, glycogen storage disease; KO, KEGG orthologs.

In our cohort, *Firmicutes*, *Bacteroidetes*, *Proteobacteria*, and *Actinobacteria* (mean abundance > 1%) were dominant in all the groups (**Figure** **3**A). However, the abundance of these phyla varied across age groups (Figure 3A,B). In particular, the relative abundance of *Bacteroidetes* decreased in all GSD subgroups, whereas *Firmicutes* and *Verrucomicrobia* significantly decreased in the GSD‐Ia and GSD‐Ib subgroups (Figure 3B). *Proteobacteria* were extended in patients with GSD, especially types Ia and Ib, whereas *Actinobacteria* were primarily increased in GSD‐no Ia, Ib, and Ia (Figure 3B). The phylum *Firmicutes* contained the most GSD‐associated microbial taxa (Figure 3C), and an overview of the bacterial taxonomic distribution at the genus level across groups and ages is summarized in Figure S3A (Supporting Information). Notably, the prevalence of certain bacteria and detectable colonization of bacteria in the healthy gut were disrupted in patients with GSD (Figure S3B,C, Supporting Information). Thirty‐one genera were significantly altered in patients with GSD compared to controls, including 18 decreased genera, whereas the remaining 13 genera were increased (Figure S4A, Supporting Information). Twenty‐eight of the 31 genera were significantly associated with GSD (Table S4, Supporting Information). At the species level, 22 species with significant changes in abundance and GSD associations were identified (Figure S4B and Table S4, Supporting Information), which included ten extended and 12 depleted species. The total abundance of the 31 genera reached 60–70% across groups, and the summed abundance of the 22 species was ≈25% (Figure 3D,E), which represented the main proportion of the gut microbiota in our cohort. In addition, patients with GSD‐Ia and GSD‐Ib exhibited more severe dysbiosis, even though inter‐subtype variations were evident (Figure 3B,D,E). In particular, most common microbial taxa,^[^ ^7^, ^18^ ^]^ such as *Akkermansia*, *Faecalibacterium*, *Bacteroides*, and *Ruminococcus*, were almost completely depleted in GSD‐Ia and‐Ib (Figure 3D,E; Figure S3B,C, Supporting Information). Conversely, potential pathogens and oral microbes such as *H. parainfluenzae*, *S. anginosus*, and *S. epidermidis* (*e*HOMD;) were present in patients with GSD, especially the type‐Ia and Ib (Figure 3E). Notably, *Bifidobacterium*, *Veillonella*, and *Lactobacillus* are maintained at high proportions during the early life of healthy children and that these bacteria expand throughout childhood and adulthood in patients with GSD (Figure 3D,E).^[^ ^19^ ^]^

Based on random forest (RF) classification, we verified the performance of the aforementioned features in characterizing the gut microbiota of patients with GSD. As expected, features from each phylogenetic level identified patients with GSD (Table S5, Supporting Information). To avoid sampling bias in model construction, tenfold cross‐validation RF models were constructed, which showed excellent efficiency (AUC 0.793–0.872 for selected features and AUC 0.811–0.892 for all features). In particular, in distinguishing GSD subgroups, especially GSD‐Ia and‐Ib, the predictive value of the models based on the five phyla or 28 genera reached high AUC values of 0.830, 0.863, 0.937, and 0.943, respectively (Figure 3F).

Next, we explored the potential interplay between the 31 genera in the control and GSD groups. The 31 genera formed a relatively balanced correlation network (66 negative and 124 positive correlations) in the healthy cohort, but were extremely imbalanced in the GSD cohort (61 negative and 124 positive correlations) (Figure 3G). Obviously, *Veillonella*, *Bifidobacterium*, *Lactobacillus*, *Enterococcus*, *Streptococcus*, *Megasphaera*, *Haemophilus* and *Turicibacter* formed a covariation network (0 negative and 19 positive correlations in the inner network), which repressed (a total of 59 negative correlations) normal bacterial colonization in patients with GSD (Figure 3G). Therefore, we quantitatively evaluated the developmental status of gut microbiota in patients with GSD using regression analysis. Both RF regression and adjusted deep neural network analyses showed disrupted microbiota for age‐Z scores (MAZ) in GSD‐no IaIb and decreased MAZ in GSD‐Ia and Ib (Figure 3H). To confirm this, 30 age‐discriminatory genera were identified using RF regression (Figure S5A, Supporting Information), 12 of which belonged to 31 genera (Figure 3D; Figure S5A, Supporting Information). There were three distinct clusters of genera with abundant changes across the ages in the GSD: stunted colonization, disrupted colonization, and persistent overgrowth (Figure S5B, Supporting Information). Notably, in the whole group, 11 of the 12 genera (*Fusobacterium* excluded) showed a tendency for abundance alterations independent of age between the GSD and control groups (Figure S5C, Supporting Information).

We further investigated the microbial functional alterations in patients with GSD and identified 73 microbial Kyoto Encyclopedia of Genes and Genomes (KEGG) pathways (kos, Level 3, L3) and 1488 KEGG orthologs (KOs) (Figure 3I,J) with both statistical abundance changes and GSD associations (Tables S6 and S7, Supporting Information). Starch and sucrose metabolism were significantly increased in patients with GSD (Figure 3I) as a result of UCCS use. However, butanoate and propanoate metabolism were abnormally decreased in patients with GSD (Figure 3I). Despite partial heterogeneity, functional alterations were more obvious in GSD‐Ia and GSD‐Ib (Figure 3J).

### 2.4 Gut Dysbiosis as a Significant Mediator Contributes to GSD Comorbidities

**Figure 4:**
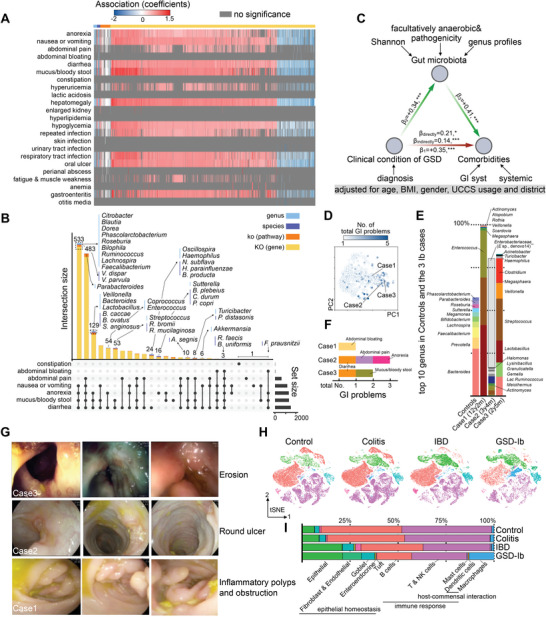
Gut dysbiosis associated with gastrointestinal (GI) phenotypes in GSD and the distinct gut microbiota and colonic epithelium profiles in three patients with GSD‐Ib. A) Heatmap showing the coefficients (association) between each gut microbial feature (taxa and function) and comorbidity. All the associations are calculated under adjustment of age, gender, district and BMI. B) UpSet plot showing the number of differential microbial features (A) associated significantly with each GI problem. C) Mediation linkages among the clinical condition of GSD, gut dysbiosis and body comorbidities. Standardized regression coefficients (β) are controlled for age, BMI, gender, UCCS usage and district. D,E) Characteristics of gut microbiota in the three patients with GSD‐Ib. Localization of gut microbiota in PCoA of the three selected patients with GSD‐Ib (D) and their genus level compositions (E). F,G) Clinical‐interview (F) and endoscopic (G) manifestations of GI system of the three selected patients with GSD‐Ib. H) tSNE plot displaying 10 subtypes, color according to different clusters. I) Bar plots exhibiting the proportion of different clusters in each group. Blocks represent different patients and are color‐coded by their derived groups. Block heights are proportional to the number of detected cells. GSD, glycogen storage disease.

We then analyzed the potential association between gut microbiota and clinical symptoms. Although there were considerable complexities (Table S8, Supporting Information), the associations between microbial features and symptoms were more focused on the gastrointestinal system, hyperuricemia, hepatomegaly, hypoglycemia, repeated infections, respiratory tract infections, oral ulcers, and gastroenteritis (**Figure** **4**A). Importantly, increased microbial features (genera, species, ko, and KO) were positively associated with the incidence of comorbidities, whereas decreased microbial features were negatively associated (Figure 4A). For example, *V. dispar* and *V. parvula* were positively associated with nausea/vomiting, anorexia, mucus/bloody stools, and diarrhea, whereas *Faecalibacterium* was negatively associated (Figure 4B; Table S8, Supporting Information). To evaluate whether gut bacterial alterations could mediate the impact of GSD on host clinical manifestations, a mediation model was constructed using the most representative gut microbial characteristics and clinical metadata. We adjusted for the covariates age, BMI, sex, daily UCCS, and district and found that the gut microbiota was a significant mediator of inner GSD and its associated comorbidities (β_indirectly_ = 0.14, *P* < 0.001) (Figure 4C).

### 2.5 Cohort Characteristics and Single‐Cell Profiling of the Colonic Microenvironment

The microbiota and colonic epithelium form a complex interplay network that is essential for colonic homeostasis. The gut microbiota of the three GSD‐Ib groups was dramatically different from that of the control (Figure 4D,E). To unveil the response of the colonic epithelium to altered microbiota in GSD‐Ib, three colonic biopsies from GSD‐Ib patients were collected and single‐cell RNA sequencing was performed. To comprehensively characterize the biological processes of GSD‐Ib, we used our previous datasets of colitis (n = 3), IBD (n = 3), and healthy groups (n = 3) as control^[^ ^20^ ^]^ (Figure S1B and Table S9, Supporting Information). Patients with GSD‐Ib showed gastrointestinal symptoms (Figure 4F) as well as endoscopic features of erosion, deep round ulceration, inflammatory polyps, obstruction, and stenosis (Figure 4G). Patients with colitis showed edematous mucosa and erythema, whereas those with IBD presented with deep linear ulcerations or cobblestone mucosa (Figure S1B, Supporting Information).^[^ ^21^ ^]^ Using scRNA‐seq, we obtained 70 490 immune cells and 19 245 non‐immune cells and partitioned them into ten groups (Figure 4H). Accordingly, macrophages, epithelial cells, fibroblasts, and endothelial cells were more abundant in the GSD‐Ib group than those in the other three groups (Figure 4H,I; Table S10, Supporting Information).

### 2.6 Typical Enteric Host‐Pathogen Interplay Mediates Specific Macrophage Activation and Accumulation in GSD‐Ib

**Figure 5:**
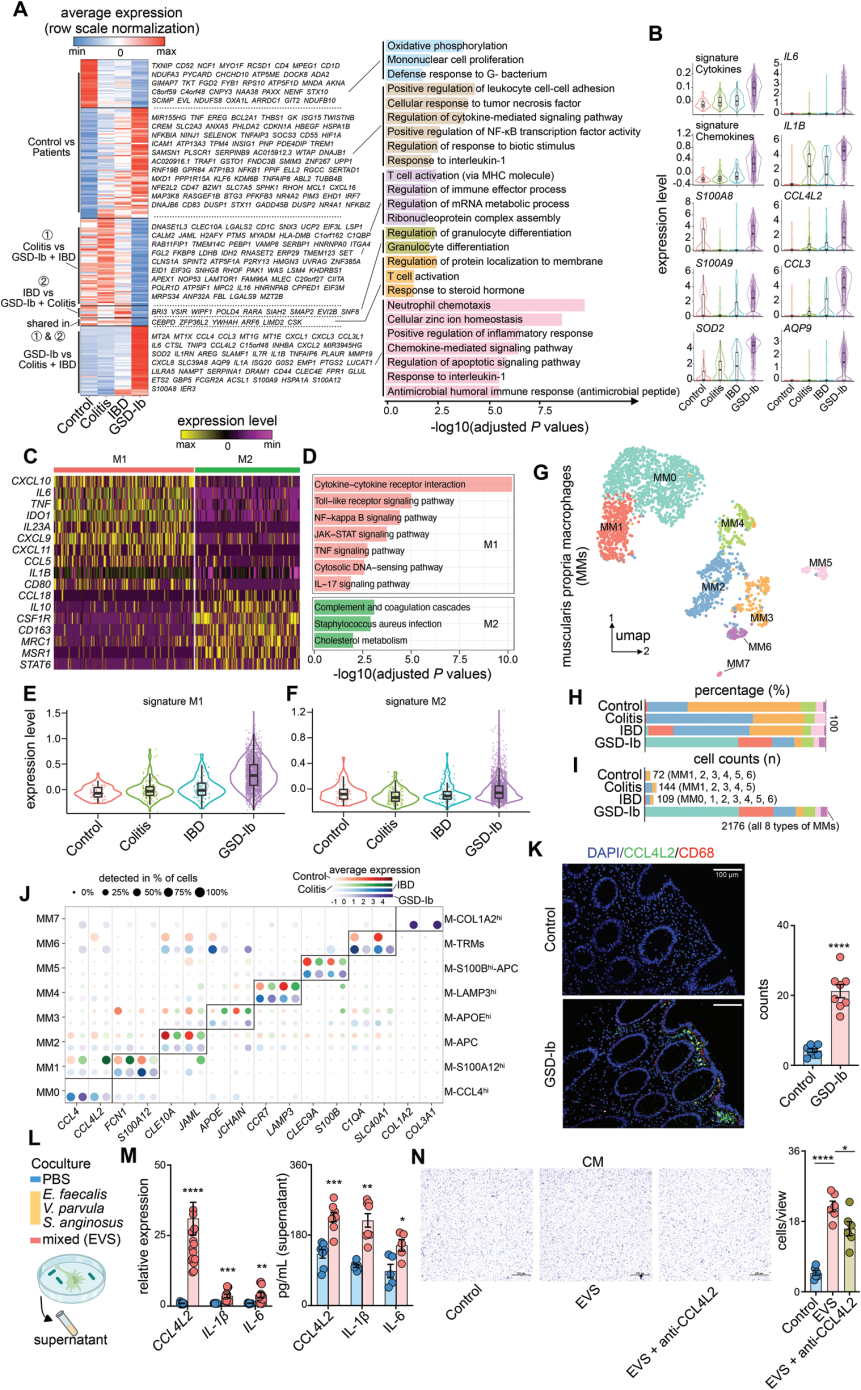
Typical enteric pathogens trigger specific macrophage activation in GSD‐Ib. A) Heatmap showing the differentially expressed genes between the groups and bar plot showing the enriched gene ontology terms. B) Violin plots and box plots showing the expression of selected signatures and differentially expressed genes in different groups. C) Heatmap showing the expression of classic genes associated M1 and M2 macrophage polarization. D) Bar plot showing the enriched KEGG pathways in M1 and M2 macrophage. E,F) Violin plots and box plots showing the expression of M1 (E) or M2 signatures (F) in different groups. G) UMAP plot displaying color‐coded macrophage subclusters. H,I) Bar chart showing the proportion (H) or cell number (I) of different subclusters in different groups. J) Dot plots showing representative DEGs between the groups across macrophage subclusters. Dot size is proportional to the fraction of cells expressing specific genes. Color intensity corresponds to the relative expression of specific genes. K) Immunofluorescent staining showing the expression levels of CCL4L2 in macrophage (n=8). *P* values were calculated by the unpaired *t* test. L,M) A model of coculture was established and the mRNA and protein level of CCL4L2 (n=18 for qPCR, n=8 for ELISA), IL‐1β (n=12 for qPCR, n=8 for ELISA), and IL‐6 (n=12 for qPCR, n=5 for ELISA) were determined in THP‐1‐derived macrophages treated with mixed indicated intestinal flora (EVS). *P* values were calculated by the unpaired *t* test. N) Transwell assay was performed to detect the effect of conditional media (CM) from THP‐1‐induced macrophages treated with and without EVS and combined with anti‐CCL4L2 on macrophages migration (n=6), *P* values were calculated by One‐ANOVA. Data are represented as mean ± SEM, ^*^ *p* < 0.05, ^**^ *p* < 0.01, ^***^ *p* < 0.001 and ^****^ *p* < 0.0001. DEG, differentially expressed gene; GSD, glycogen storage disease.

KEGG pathway annotation of the differentially expressed genes (DEGs) in macrophages revealed a significant enrichment of gene sets regulating the antimicrobial humoral immune response and chemokine‐mediated signaling pathways in GSD‐Ib (**Figure** **5**A; Table S11, Supporting Information). The upregulated genes in the GSD‐Ib group were primarily involved in environmental information processing and human diseases (Figure S6A, Supporting Information). Typical genes participating in chemotaxis (*CCL4L2*), cytokine signaling (*IL6* and *IL‐1β*), and bacterial infections (*Shigellosis*), are shown in Figure 5B and Figure S6B (Supporting Information). Macrophages were then sub‐grouped into inflammatory M1 and anti‐inflammatory M2 macrophages (Figure 5C,D) via the macrophage polarization‐scaled signature (Figure S6C, Supporting Information). Both M1 and M2 macrophages were upregulated in GSD‐Ib (Figure 5E,F), indicating a mix of M1/M2 macrophage activation. However, this classification profiled only 20.6% of all macrophages (Figure S6D, Supporting Information). To gain further insight, the macrophages were clustered into eight subsets and designated as MM0 to MM7 (Figure 5G; Table S12, Supporting Information). Importantly, MM0 was the most abundant subset of GSD‐Ib (Figure 5H,I), with the highest expression of chemokines *CCL4* and *CCL4L2* (Figure 5J,K; Figure S6E,F, Supporting Information), as well as pro‐inflammatory genes such as *IL‐1B*, *IL‐6*, and *CXCL8* (Table S12, Supporting Information).

We selected three GSD‐related species (Figure 3D,E), E. *faecalis*, *V. parvula*, and *S. anginosus*, to stimulate the macrophages. The expression of CCL4L2, which was significantly increased at mRNA and protein level in macrophages exposed to *E. faecalis*, *V. parvula*, and *S. anginosus*, as well as mix infection (EVS), does not impact the expression of IL‐1*β* and IL‐6 (Figure 5L,M; Figure S7A‐E, Supporting Information). Conditional medium (CM) from the EVS infection induced significant chemotaxis of macrophage migration in comparison with that of the control, which was partly attenuated by CCL4L2 neutralization (Figure 5N).

### 2.7 Distinct Epithelial Cells Profiles Related to Infections and Epithelial Barrier Functions

**Figure 6:**
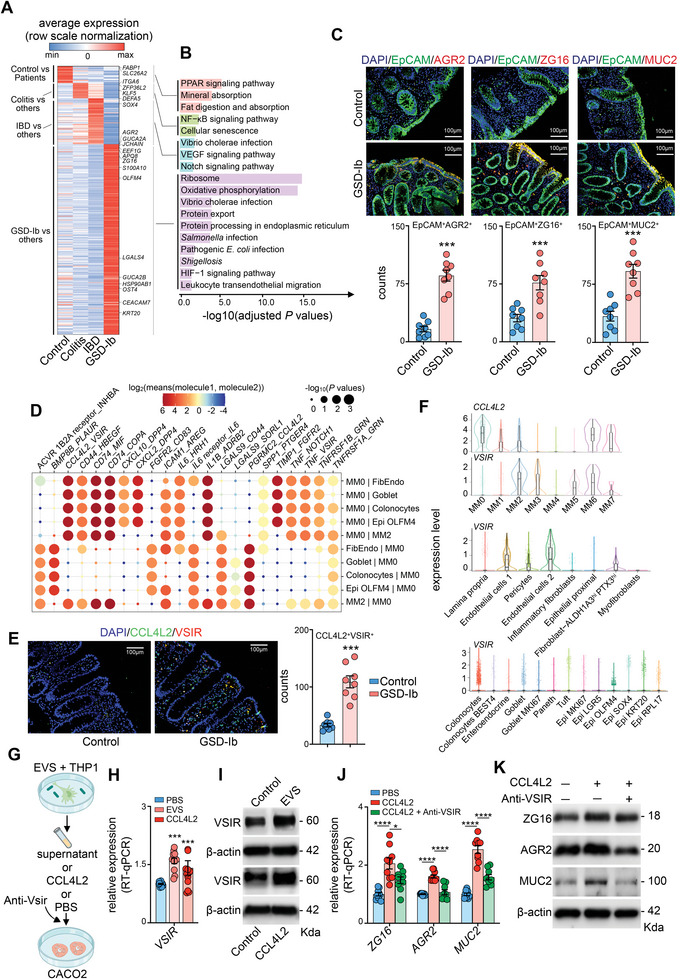
Distinct epithelial and endothelial cells profiles related to infections and CCL4L2‐VSIR pair. A) Heatmap showing the differentially expressed genes between the groups. B) Bar plot showing the enriched KEGG pathways in different groups. C) IF showing AGR2, ZG16, and MUC2 expression in intestinal epithelial cells (n=8). *P* values were calculated using unpaired t‐tests. D) Predicted interactions between MM0 macrophages and other cell types. E) IF showing the CCL4L2/VISR pair in different groups. *P* values were calculated by the unpaired t test (n=8). F) The expression of CCL4L2 and VSIR in macrophages, epithelial cells, fibroblasts and endothelial cells. G,I) The schematic flow of coculture, and qPCR (H: n=12) and WB (I: n=3) analysis of VSIR was performed. *P* values were calculated by the unpaired t test. J,K) real‐time PCR (J: n=9) and WB (K: n=3) were used to detect ZG16, AGR2, and MUC2 in CaCO2 cells treated as indicated. *P* values were calculated using one‐way ANOVA. Data are represented as mean ± SEM, ^*^ *p* < 0.05, ^**^ *p* < 0.01, ^***^ *p* < 0.001 and ^****^ *p* < 0.0001.

Gene sets regulating ribosomes, oxidative phosphorylation pathways, and anti‐pathogen responses were specifically enriched in GSD‐Ib epithelial cells (**Figure** **6**A,B). Typical DEGs in epithelial cells were *AQP8*, *ZG16*, *GUCA2C*, *SLC26A3*, and *AGR2* (Figure 6C; Figure S8A and Table S13, Supporting Information), which are responsible for absorption, secretion, metabolism,^[^ ^22^ ^]^ epithelial barrier integrity,^[^ ^23^ ^]^ pH homeostasis,^[^ ^24^ ^]^ and mucus barrier function.^[^ ^25^ ^]^ Epithelial cells were subgrouped into 13 subsets, and six clusters of colonic stem cells were identified (Figure S8B and Table S14, Supporting Information). Importantly, OLFM4^+^ epithelial cells, which are involved in digestive diseases and active lesions of Crohn's disease,^[^ ^26^ ^]^ were mainly present in the GSD‐Ib group compared with the other groups (Figure S8B, Supporting Information). Genes regulating major histocompatibility complex (MHC) molecules and pathogenic bacterial infections were enriched in OLFM4^+^ epithelial cells and colonocytes (Figure S8C, Supporting Information).

To examine the molecular connection between macrophages and tissue‐resident cells, immune‐related ligand‐receptor pairs were used to gain insight into potential intercellular interactions. Cell–cell interactions were significantly enhanced in GSD‐Ib cells, particularly in macrophages (Figure S8D, Supporting Information). In particular, the ligands *CCL4L2*, *CD74*, *IL1B*, and *TIMP1* were highly expressed in MM0 macrophages and interacted with the receptors, *VSIR*, *MIF*, *COPA*, *ADRB2*, and *FGFR2* expressed in goblets, colonocytes, and *OLFM4* ^+^ cells, respectively (Figure 6D). The CCL4L2‐VSIR pair “don't eat me” signals was significantly correlated with T cell exhaustion,^[^ ^27^ ^]^ which mediated macrophage interaction with goblet, and colonocytes (Figure 6D).

In the intestinal tissues of patients with GSD‐Ib, significant co‐localization of CCL4L2 and VSIR was observed (Figure 6E), and the corresponding cellular expression profiles are detailed in Figure 6F. In in vitro experiments, conditioned media (CM) from THP‐1‐derived macrophages treated with EVS or CCL4L2 (200 ng mL^−1^) induced the overexpression of *VSIR* in CaCO2 cells (Figure 6G–I). CCL4L2 stimulation enhanced *MUC2*, *AGR2*, and *ZG16* expression at both the mRNA and protein levels in CaCO2 cells, whereas the process was reversed by the addition of neutralizing antibodies against VSIR (Figure 6J,K). Additionally, anti‐VSIR had the same effect on MUC2, ZG16, and AGR2 expression in the EVS‐treated coculture system (Figure S8E, Supporting Information).

### 2.8 Blockade of CCL4L2/VSIR Aggravated EVS‐Derived IBD In Vivo

**Figure 7:**
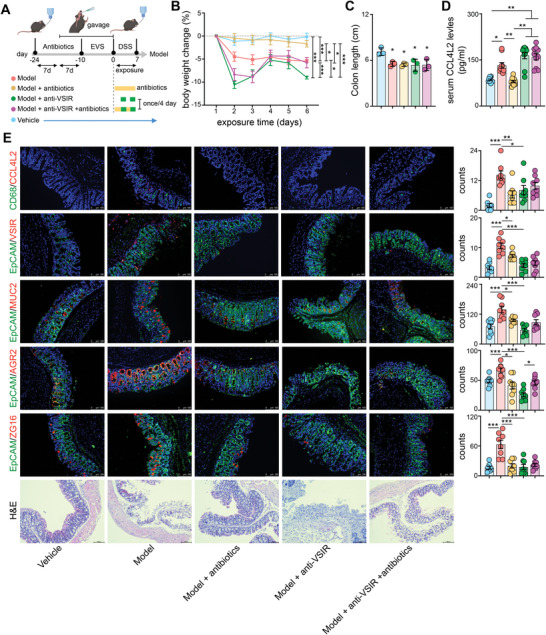
Blockade of CCL4L2/VISR aggravated EVS‐derived IBD‐like conditions in vivo. A) Schematic overview of DSS‐induced model with or without antibiotics/anti‐VISR antibody. B) Mouse body weight was monitored for indicated times after DSS treatment. *P* values were calculated by the One‐ANOVA. C) Statistical analysis of colon length in indicated group that were determined by the one‐way ANOVA (n=3). D) Serum CCL4L2 was measured in indicated group by ELISA assay. *P* values were calculated by the One‐ANOVA (n=10). E) Immunofluorescent staining showing the expression levels of CCL4L2, MUC2, EpCAM, AGR2, ZG16. *P* values were calculated by the One‐ANOVA (n=8). Data are represented as mean ± SEM. ^*^ *P*<0.05, ^**^ *P*<0.01, ^***^ *P*<0.001 and ^****^ *P*<0.0001. DSS, dextran sodium sulfate; EVS, *E. faecalis, V. parvula, and S. anginosus*; IBD, inflammatory bowel disease.

To further confirm the role of the CCL4L2/VSIR axis in gut dysbiosis‐derived IBD in vivo, selected bacterial transplantation (SBT) was performed in C57BL/6 mice (**Figure** **7**A). After removing native gut microbiota (Figure S9A, Supporting Information), the mice received EVS transplantation by oral gavage for 10 days (Figure S9B,C, Supporting Information), colitis was induced by adding 2.5% dextran sodium sulfate (DSS) to drinking water for 7 days. Consistently, the gut microbiota was the major driver of the variable response to DSS‐induced colitis,^[^ ^28^ ^]^ as elimination of the gut microbiota (model + antibiotics) offset weight loss (Figure 7B). Although no significant difference in colon length was found, anti‐VSIR injection aggravated body weight loss in comparison with the model group, which was significantly reversed in the model+anti‐VSIR+antibiotics group (Figure 7B,C).

Antibiotic treatment significantly decreased EVS gavage‐induced mouse serum CCL4L2 elevation, whereas anti‐VSIR showed a completely opposite trend, similar to the typical adverse reaction, cytokine storm, in clinical applications of anti‐PD1 therapy^[^ ^29^ ^]^ (Figure 7D). Immunofluorescent staining showed that MM0 macrophages were enhanced in the model group, but decreased after administration of antibiotics or anti‐VSIR injection, which further led to significant downregulation of MUC2, AGR2, and ZG16 expression in the intestinal epithelial cells of the model group, triggering mucosal injury (Figure 7E).

Collectively, our findings suggest that EVS‐boosted MM0 macrophages boosted by EVS could mediate colonic intestinal epithelial homeostasis by modulating the CCL4L2‐VSIR axis. Thus, targeting the CCL4L2‐VSIR pathway can be exploited for broad applications in colitis and IBD.

## 3 Discussion

Based on our multiregional population‐based cohort, we showed that patients with GSD experienced varying degrees of digestive symptoms, including specific colonic endoscopic characteristics, such as single or multiple scattered deep round ulcers, inflammatory pseudo‐polyps, obstructions, and strictures. We reported unique gut microbiota immaturation in patients with GSD and concluded that gut dysbiosis was caused mainly by GSD rather than by external factors, such as UCCS.^[^ ^13^ ^]^ We further explored the mediating role of gut microbiota in GSD‐associated IBD using cohort metadata analysis, scRNA‐based colonic mucosal profiling, and animal model studies. Accordingly, enteric pathogen outgrowth and a stunted gut microbiota promote the activation and accumulation of macrophages. These inflammatory macrophages are characterized by high expression of *CCL4L2*, mediate inflammatory effects on epithelial cells, and eventually contribute to IBD in patients with GSD‐Ib.

Specific patterns of gut dysbiosis were observed in patients with a GSD of 1) Most of the decreased microbial taxa in GSD were common and considered functional microbes that colonized the healthy human gut; 2) Extended microbial taxa in GSD are rare or very low in abundance in the healthy population, and some are potential oral pathogens related to systemic infections; 3) significant and persistent gut microbiota immaturity is specifically present in patients with GSD; 4) high heterogeneity in the gut microbiota exists among patients with GSD. Accumulating evidence has shown that short‐chain fatty acids, which are products of bacterial fermentation, contribute to the gut luminal pH balance.^[^ ^30^ ^]^ Butyrate has been reported to enhance epithelial oxygenation to favor a low‐oxygen microenvironment in the colon^[^ ^31^ ^]^ and inhibit pathogen colonization.^[^ ^32^ ^]^ However, its main producers, such as *Bacteroides*, *Faecalibacterium*, and *Ruminococcus*, as well as related metabolic pathways were significantly decreased in GSD, which may have led to the extension of facultative anaerobic pathogens.

In contrast, an increased number of bacteria such as *Streptococcus* have been shown to induce gut damage through gasdermin A‐dependent pyroptosis.^[^ ^33^ ^]^ Members of *Enterococcus* have shown proinflammatory effects.^[^ ^34^ ^]^ In addition, *Veillonella* has been found to be extended in IBD^[^ ^35^, ^36^, ^37^ ^]^ and *V. parvula* induces inflammation through nitrate production.^[^ ^38^ ^]^ Furthermore, the combination of *Veillonella* and *Streptococcus* inhibited the biosynthesis of *IL‐12p70* and subsequently enhanced *IL‐8*, *IL‐6*, and *TNFα* inflammatory responses.^[^ ^39^ ^]^ Altogether, this suggested that interactions among bacteria in GSD formed an independently operated mutual supplied system, and gut dysbiosis induced intestinal epithelial dysfunction in patients with GSD possibly through bacterial “group crime”.

Crosstalk between gut‐resident immune cells and the epithelium is essential for gastrointestinal homeostasis, regulation of antigen sensitization, prevention of infection, and development of IBD.^[^ ^40^ ^]^ Recently, the taxonomy of inherited monogenic IBD has included *SLC37A4* and *G6PC3*.^[^ ^41^ ^]^ This finding suggests that GSD‐associated IBD are specific forms of IBD. In this study, we discovered that GSD‐associated IBD have distinct characteristics compared to typical IBD, in particular, complex endoscopic manifestations and their special deconstruction: amount of macrophage accumulation and epithelial cell proliferation. Etiologically, the activation of mucosal macrophages by enteric pathogenic bacteria leads to an inflammatory environment in the gastrointestinal (GI) tract. In GSD‐Ib, a unique group of macrophages is induced by pathogenic bacteria and express high levels of the chemokine CCL4L2. CCL4L2 contributes to inflammation and activates the CCL4L2‐VSIR signaling pathway in intestinal epithelial cells. As a result, the expression of mucosal proteins, such as AGR2 and ZG16, increases in epithelial cells, promoting the repair of mucosal damage.

Notably, the activation of the CCL4L2‐VSIR pathway contributes to T cell exhaustion in severe cytokine storms, which is associated with poor prognosis in COVID‐19.^[^ ^27^ ^]^ Targeting CCL4L2‐VSIR could inhibit various inflammatory diseases, autoimmune diseases, and tumor metastasis.^[^ ^42^, ^43^, ^44^, ^45^ ^]^ Here, we revealed that abnormal activation of the CCL4L2‐VSIR axis in the GI tract ultimately drives non‐canonical IBD in GSD‐Ib. However, the specific mechanisms by which infection regulates CCL4L2 expression, and how CCL4L2 modulates epithelial homeostasis, remain unknown. Additionally, the observed interaction between CCL4L2 and VSIR mainly occurred between macrophages and other immune cells. Limited knowledge about VSIR ligands impedes progress in the use of VSIR for treatment. Our findings introduce a novel target for diagnosing and treating GSD‐Ib, and potentially GSD‐associated IBD, as well as provide evidence for research related to VSIR.

## 4 Conclusion

In summary, we provide insights into the pathogenesis of GSD‐associated IBD by combining microbiota profiling and the single‐cell transcriptional landscape of colonic mucosae. This indicates that the disturbed balance between the microbiota, immune cells, and epithelial cells results in the activation of the CCL4L2‐VSIR pathway, which plays a crucial role in protecting the intestinal mucosa in individuals with GSD‐Ib.

## 5 Experimental Section

### Ethics Declarations

This study was approved by the Medical Ethics Committee of Guangdong Province Peoples Hospital (ID:202205303) and Guangzhou Women and Children's Medical Center (ID:201941701) and was conducted in accordance with the International Ethical Guidelines for Research Involving Human Individuals, as stated in the Helsinki Declaration. Written informed consent was obtained from all the participants or their legal guardians.

### Participants and Sample Collection

Gut microbiota A total of 477 participants from 23 provinces in China were recruited for clinical diagnosis, treatment, or physical examination. All participants and their guardians provided informed consent for stool sample collection and trial participation (Table S1, Supporting Information).

Patients with GSD: A total of 150 participants with a clinical diagnosis of GSD (aged 1–26 years) were recruited. GSD was defined based on whole‐genome/exome sequencing or panel‐based next‐generation sequencing (NGS) as described in the epidemiological survey section. The GSD group included seven types of GSD: type Ia (n = 58, 38.67%), Ib (n = 39, 26.00%), III (n = 21, 14.00%), IX (n = 20, 13.33%), VI (n = 7, 4.67%), IV (n = 3, 2.00%), and 0 (n = 2, 1.33%). All participants were reassessed in our outpatient ward or through telephone interviews (with nonlocal patients) by our clinicians.

Family controls: A total of 137 healthy linear relatives of patients with GSD were recruited, of which 82 were parents and 51 were siblings (four participants were undeclared).

Unrelated control: 190 healthy individuals aged 1–9 years from seven provinces of China (mainly Hunan, Shandong, and Zhejiang) were recruited as unrelated controls. The group was set to supplement the control population distribution in the age brackets in which patients with GSD were mainly distributed, and to analyze the impact of family factors (such as living environment, eating habits, and genetic background) on individual gut microbiota.

All the metadata considered in this study are listed in Table S2 (Supporting Information). To register the information, each sample was scored as 1 (yes) or 0 (no) for each factor. Comorbidities, such as gastrointestinal problems, sleep complaints, and immune abnormalities, refer to body conditions that occurred two weeks prior to sampling. None of the participants had received any antibiotics two weeks prior to sampling. The use of antibiotics and probiotics one month prior to sampling was also recorded.

Cohort for single‐cell Profiling: Colonic mucosal tissue was obtained from three individuals diagnosed with GSD‐associated IBD who underwent colonoscopy between October 2019 and April 2020 (n = 3). The colitis (n = 3), IBD (n = 3), and control (n = 3) groups were included in this cohort using data from previous studies.^[^ ^6^ ^]^

GSD‐associated IBD diagnosis criteria: patients diagnosed with GSD‐Ib based on whole‐genome/exome sequencing or panel‐based NGS, gastrointestinal symptoms for more than two weeks, and endoscopic features, including mucosal erosions, single or scattered multiple deep round ulcers, inflammatory polyps, obstruction, and stenosis.

Clinical severity (total disease severity score) was determined according to our previous criteria^[^ ^20^ ^]^ which modified the Pediatric Crohn's Disease Activity Index criteria^[^ ^46^ ^]^ (Table S15, Supporting Information). Clinical phenotypes included vomiting, abdominal pain, stool frequency and consistency, blood in the stool, perirectal disease, and World Health Organization weight‐for‐length Z‐score. Blood analysis was performed on the day of colonoscopy to determine the hemoglobin and albumin levels.

Endoscopic activity of the colonic tissue was determined by experienced gastrointestinal endoscopists using the modified Mayo endoscopic score criteria.^[^ ^47^, ^48^ ^]^ Endoscopic biopsies were obtained from five sites: the ileocecum and ascending, transverse, descending, and rectosigmoid colon. Histological activity was determined according to the Geboes score (GS)^[^ ^49^ ^]^ by gastrointestinal pathologists blinded to the endoscopic severity scores. The grades were as follows: 0, normal histological appearance; 1) mild to moderate structural abnormality and infiltration of eosinophils or neutrophils in the lamina propria; 2) marked infiltration of eosinophils or neutrophils in the lamina propria and/or presence of neutrophils in the epithelium; and 3) crypt destruction, erosions, or ulcerations. GS was assigned to biopsies from each colonic segment, and the highest score (the most inflamed segment by histology) was used as the histological score for each patient.

### Mice

Weight‐matched 8–10‐week‐old male C57BL/6 mice (22–25 g) were purchased from DK Pharmaceutical (Guang Dong) Co., Ltd. and fed regular food and water before the experiments in individually ventilated cages in a specific pathogen‐free barrier unit. Animal studies were approved and conducted in accordance with the guidelines of the Institutional Animal Care and Use Committee of Guangdong Provincial People's Hospital (resolution number: KYN202205303).

### Cell Lines

Intestinal epithelial cells, CaCO2 and human THP‐1 monocytes, were obtained from the American Type Culture Collection and cultured at 37 °C in 5% CO_2_ at 100% humidity in Roswell Park Memorial Institute‐1640 medium with 10% (v/v) FBS and 1% (v/v) penicillin/streptomycin.

### Fecal Sample Collection and DNA Extraction

Fecal samples from each participant were collected at home, outpatient clinic, or ward by trained guardians or qualified clinicians. In brief, sufficient fresh stools were removed into the storage kit, fully dissolved in the protective solution (Zhejiang Hangzhou Equipment Preparation NO: 20190682) within 3 min after defecation, and then transferred to a −80 °C domestic refrigerator. For samples collected at home, storage kits containing dissolved stools were sent to our outpatient clinic (< 20 °C) within 2 days of collection by guardians and then stored at −80 °C until further processing. Pipes with damage, leakage, or insufficient dissolved stool were excluded.

Total microbial genomic DNA was extracted using the GHFDE100 DNA isolation kit (Chinese national invention patent NO: ZL201511009389.7 also with the Zhejiang Hangzhou Equipment Preparation: 20190952) according the manufacturer's instructions. The extracted DNA was quantified and qualitatively analyzed using a NanoDrop ND‐1000 spectrophotometer (Thermo Fisher Scientific, Waltham, MA, USA) and agarose gel electrophoresis. Samples with incomplete or undetectable main DNA bands (15 kb) were excluded from analysis.

### PCR Amplification, Quantification and *16S* rRNA Gene Sequencing

Primers with 7‐bp sample‐specific barcodes were incorporated for multiplex sequencing. PCR amplification of the bacterial *16S* rRNA gene V4 region was performed using the forward primer 515F (5ʹʹ‐GTGCCAGCMGCCGCGGTAA‐3ʹ) and reverse primer 806R (5ʹ‐GGACTACHVGGGTWTCTAAT‐3ʹ). Sample‐specific paired‐end 7‐bp barcodes were incorporated into TrueSeq adaptors for multiplex sequencing. The PCR components contained 25 µL of Phusion High‐Fidelity PCR Master Mix, 3 µL (10 µm) of each forward and reverse primer, 10 µL of the DNA template, 3 µL of DMSO, and 6 µL of ddH_2_O. The following cycling conditions were used: initial denaturation at 98 °C for 30 s followed by 25 cycles of denaturation at 98 °C for 15 s, annealing at 58 °C for 15 s, and extension at 72 °C for 15 s and a final extension of 1 min at 72 °C. PCR amplicons were purified using Agencourt AMPure XP Beads (Beckman Coulter, Indianapolis, IN, USA) and quantified using a PicoGreen dsDNA Assay Kit (Invitrogen, Carlsbad, CA, USA). After quantification, the amplicons were pooled in equal amounts, and 2 × 150 bp paired‐end sequencing was performed using the Illlumina NovoSeq6000 platform at GUHE Info Technology Co., Ltd. (Hangzhou, China).

### Quality Control and Bioinformatics Analysis of Gut Microbiota Data

Barcode‐matched raw sequences were assigned to the corresponding samples as valid sequences. The inferior sequences were filtered out according to the following criteria: i) sequences with <150 bp length or <20 average Phred score; ii) sequences that contained ambiguous bases or >8 bp mononucleotide repeats.^[^ ^50^, ^51^ ^]^ A total of 60259799 clean reads were obtained (126330/sample).

Qualified paired‐end reads were blasted, dereplicated (–derep_fulllength), clustered (–cluster_unoise) and chimera detected (–uchime3_denovo) using VSEARCH (V2.4.4)^[^ ^52^ ^]^ against the SILVA138 database^[^ ^53^ ^]^ and then assembled into operational taxonomic units (OTU) with sequence similarity ≥ 97% using the quantitative insights into microbial ecology (QIIME2, v2020.6) pipeline.^[^ ^54^ ^]^ OTUs containing less than 0.001% of the total sequences across all samples were discarded. To minimize differences in sequencing depth across samples, an averaged, rounded, and rarefied OTU table was generated by averaging 100 evenly resampled OTU subsets at 90% of the minimum sequencing depth for further analysis.

OTU‐level alpha diversity, such as Chao1 richness, abundance‐based coverage estimator, Shannon, and Simpson indices of each sample were calculated using the OTU Table in QIIME2. Beta diversity analysis was performed using Bray–Curtis dissimilarity metrics and visualized via principal coordinate analysis based on OTU‐level compositional profiles.^[^ ^55^ ^]^ Microbial functions were predicted by phylogenetic investigation of communities using the reconstruction of unobserved states (PICRUSt) based on high‐quality sequences.^[^ ^56^ ^]^ The relative abundances of KEGG pathways and KOs were calculated by adding the relative abundances of genes belonging to the same pathways or KOs. Statistical testing was performed using the limma package in R with the Benjamini–Hochberg False Discovery Rate (FDR) method. Community‐level properties of the gut microbiota, such as potential pathogenicity, facultative anaerobic, and anaerobic, were predicted using Bugbase. The body‐site origins of the microbes were identified according to *e*HOMD.

### Multivariable Analysis

Covariates and significance of each factor on gut microbiota variations from metadata were determined by EnvFit^[^ ^17^ ^]^ based on the NMDS with Bray–Curtis dissimilarity, and the multivariate linear modeling system MaAslin2^[^ ^19^ ^]^ was used to calculate the association between selected microbial features and clinical factors for the fixed effects of age, BMI, sex, and district, in the “vegan” R package. Significance values across all associations were adjusted using the Benjamini–Hochberg FDR method.

### Random Forest Classification

RF analysis was applied to discriminate samples from different groups using the R package “randomForest” with 1000 trees and all default settings off.^[^ ^57^ ^]^ AUC of the models were calculated based on the inputted relative abundance files of the given microbial features with parameters “no validation” or “tenfold cross‐validation.”

### Analysis of Microbiota Development Status

RF regression and adjusted convolutional neural networks (CNNs) classification were performed to evaluate the microbiota age of each patient quantitatively.

The relative abundances of bacterial taxa of the microbiota at the genus level in the time‐series profiling of both the family and unrelated controls were extracted and regressed against their chronological age by RF regression. To obtain the best variance explanation related to chronologic age, parameters of the RF regression was optimized as: R package “RF,” ntree 500, using default mtry. The report of ranked lists of important genera in order of reported feature importance was output after 100 iterations of the algorithm, and 31 were selected to map the developmental spectrum of the gut microbiota among groups based on all control patients included in the model. The explanatory variance for this model was 47%. Furthermore, healthy patients were subdivided into age < 3, 3–9, 10–18, and >18 years old to build the corresponding models without any further parameter adjustment, which were then applied to patients with age‐matched GSD. The variance explanations for the models were 57%, 51%, 43%, and 39%, respectively.

Adjusted multiclass CNNs classification models were used to further confirm age‐related microbiota alterations in patients with GSD.

The abundances of 75489 microbial taxa at the OTU level of 23107 healthy patients (aged one week to 102 years) from the GUHE self‐sequence cohort was used as the training set. To form a 256 × 256 pixel diagram representing the overview of OTU abundance for later CNNs classification, three numerical value matrices were generated from the selected 65536 OTUs (the most abundant OTUs), genus level (summed abundance of the contained OTUs), and OTU‐level abundances, and the correlations between the abundance of each genus. Additionally, only correlations greater than 0.75 were involved, and abundances of OTUs were logarithmically transformed (ranging from −1 to 1) and then normalized (ranging from 0 to 255). Genera were positioned according to their abundance and correlations with specific paired genera, ranging from high to low. After positioning, OTUs belonging to the corresponding genera were shaded according to their normalized abundances and output in a 256×256 pixel diagram. To maximize model efficiency, 23 107 patients were divided into 52 subgroups according to their physiological age: 0–1 years monthly, 1–2 years every two months, 2–4 years every three months, 4–10 years yearly, 10–22 years every two years, 22–40 years every three years, 40–70 years every five years, and 70–102 years every eight years; each subgroup contained at least 100 patients. The corresponding diagrams were input into the CNNs against the sample physiological age using TensFlow 1.13.1, trained by ResNet50^[^ ^58^ ^]^ and finally the three predicted ages with the highest incidence were output. The microbiota age of each patient was calculated as the median of the three predicted ages. The performance of the adjusted CNNs model was evaluated in our validation set (another 8706 samples from GUHE) using the MAE, and the R^2^ value was 0.9554.

The MAZ were calculated according to the protocol proposed by Subramanian et al.^[^ ^59^ ^]^:

MAZ = (microbiota age–median microbiota age of healthy children of the same chronological age) / (s.d. of microbiota age of healthy children of the same chronological age)

### Mediation Model

The mediation model was constructed using the AMOS (V2020) software. For the model construction, the input factor‐type indicators were numerically converted. The diagnosis of GSD or comorbidities was converted from yes to 1 and no to 0, respectively, while for females it was converted from 0 to 1. Districts were transferred according to the number of participants. Age, BMI, sex, UCCS usage, and district were covariates of the GSD clinical condition. To avoid bias by too many included factors in proving the mediating variate gut microbiota, the abundance of the 28 genera was combined to output a predicted value describing whether the diagnosis of GSD of the patient was through logistic regression.^[^ ^60^ ^]^ The AUC of the logistic regression classifier was 1. The simulation exercises were bootstrapped 5000 times to confirm the results, and the performance of the mediation model was: χ^2^ = 46.104, df = 32, CFI = 0.993, TLI = 0.985, RMSEA = 0.030, and SRMR = 0.019. Similar results were obtained for all 28 genera in the mediation model.

### RNA‐seq Library Preparation for 10x Genomics Single‐Cell 5ʹ Sequencing

Single‐cell 5ʹ RNA sequencing libraries for each of the 12 samples were separately constructed according to the protocols of the Chromium Single Cell 5ʹ Library kit (10x GENOMICS). Briefly, a single‐cell suspension mixed with RT‐PCR master mix was loaded with partitioning oil and nanoliter‐scale gel beads into a single‐cell 5ʹchip according to the manufacturer's instructions. To generate a single multiplexed library, RNA transcripts were uniquely barcoded within single cells and reverse transcribed into barcoded cDNAs, which was then purified, amplified, end‐repaired, and ligated using Illumina adapters. All libraries were sequenced on an Illumina NovaSeq 6000 platform to generate 150 bp paired‐end reads.

### Single‐cell RNA‐Seq Data Processing

The Cell Ranger toolkit (v6.0.1) provided by 10x Genomics was used to align reads and generate a gene‐cell unique molecular identifier (UMI) matrix using the human reference genome GRCh38. DoubletFinder^[^ ^61^ ^]^ was used to identify and remove potential doublets. Cells were selected according to the following criteria: > 600 UMI counts; < 20000 UMI counts; > 400 genes; < 5000 genes; and < 25% UMIs derived from the mitochondrial genome. After quality control, 89735 high‐quality cells were obtained.

The Seurat R package^[^ ^62^, ^63^ ^]^ (v3.2.3) was used to perform the unsupervised clustering of single cells using the UMI count matrix as the input. First, the gene expression matrices were normalized to the total UMI counts per cell, multiplied by the scale factor (10 000), and then natural log‐transformed. The top 2000 highly variable genes were selected using the FindVariableFeature function in Seurat. The resulting gene expression matrix was scaled and individualed to principal component analysis. The first 20 principal components were used for cluster analysis with suitable resolution and visualization using a uniform manifold approximation and projection algorithm. The FindAllMarkers function in Seurat was used to identify marker genes for each cluster using the default parameters. The main cell types were assigned based on the expression of known cell type markers. Harmony (v1.0)^[^ ^64^ ^]^ was applied for batch effect correction before the clustering analysis. Macrophages, epithelial cells, and other stromal cells were clustered using the same approach, to generate a more refined map.

### DEG Analysis and Enrichment Analysis

FindAllMarkers function was used to find DEGs between each subtype, with only.pos = TRUE, an average log‐scaled fold change > 0.5 and adjusted *P* value < 0.01 (Wilcoxon rank sum test). Gene specificity was determined by restricting the value of pct.1 minus pct.2. The DEGs of each subtype were chosen to perform Gene Ontology (GO) and KEGG enrichment analyses using the clusterProfiler^[^ ^65^ ^]^ package (v4.0.5), and the results were visualized using the ggplot2 R package.

The AddModuleScore function in the Seurat package was used to calculate the gene set scores for each cell, which calculated the average expression levels of each program at the single‐cell level subtracted from the aggregated expression of the control feature sets. M1 and M2 signatures were calculated for all macrophages and scaled. M1 subpopulations were defined based on M1 signature > 1.1 and M2 signature < 0.9. M2 subpopulations were defined based on M1 signature < 0.9 and M2 signature > 1.1.

### Cell–Cell Communication Analysis

Cell–cell interactions based on the expression of known ligand–receptor pairs in different cell types were inferred using CellPhoneDB.^[^ ^66^ ^]^ This tool evaluates the impact of ligand/receptor interactions based on ligand expression in one cell type and the corresponding receptor expression in another. This method permuted the change in cell‐type labels for each cell 1000 times to calculate the significance of each pair. The *P* value was calculated using the proportion of the mean value for specific receptor–ligand pairs compared to a randomly permuted mean distribution. Additionally, cell types with very small percentages were excluded to obtain more accurate and realistic results.

### Immunofluorescent Staining

As described in our previous study,^[^ ^20^ ^]^ paraffin‐embedded sections of colonic biopsies were deparaffinized, antigen retrieved, and blocked with goat serum at room temperature (23 ± 2 °C) for 1 h, followed by incubation with primary antibodies overnight in a wet chamber at 4 °C in the dark. After washing with PBS, the sections were stained with Alexa‐647‐ or Alexa 488‐conjugated secondary antibodies for 1 h at room temperature in the dark and mounted on coverslips with Prolong Gold Antifade reagent (after staining the nuclei with DAPI). The slides were imaged using Leica X image analysis software (Leica, Hamburg, Germany) and ImageJ software (National Institutes of Health, MD, USA). The antibodies used for Immunofluorescent staining listed as followed: rabbit anti‐VSIR (54979, Cell Signaling Technology, Shanghai, China; 1:200), mouse anti‐CCL4L2 (H00388372‐M01, Novus, Centennial, CO, USA; 1:25), rabbit anti‐MUC2 (A14659, Abclonal, Hubei, China; 1:100), rabbit anti‐ZG16 (17397‐1‐AP, Proteintech, Hubei, China; 1:50), rabbit anti‐AGR2 (12275‐1‐AP, Proteintech; 1:100), mouse anti‐CD68 (YM3050, Immunoway, Plano, TX, USA; 1:100), mouse anti‐EpCAM (YM6053, Immunoway; 1:100). Goat anti‐rabbit IgG H&L (Alexa Fluor 647) (ab150079, Abcam, Waltham, MA, USA; 1:200) and goat anti‐mouse IgG H&L (Alexa Fluor 488) (ab150113, Waltham, MA, USA; 1:200) were used.

### Enzyme‐Linked Immunosorbent Assay

Quantitative analysis of CCL4L2, IL‐1*β*, and IL‐6 in the samples was carried out using an ELISA kit according to the manufacturer's instructions. Concentrations were quantified using the corresponding standard curves. Each sample was analyzed in duplicate.

### Real‐Time Quantitative PCR (RT‐qPCR) Amplification

Total RNA was obtained using the RNAeasy Animal RNA Isolation Kit with Spin Column (R0027, Beyotime, Shanghai, China) and reverse‐transcribed into cDNA using the PrimeScript RT Master Mix Kit (RR036A, Takara, Beijing, China). All gene transcripts were amplified by PCR using the TB GreenPremix Ex Taq II Kit (RR820A, Takara) on a CFX96 Real‐Time PCR Detection System (Bio‐Rad). The mRNAs levels of the target genes and the housekeeping gene (GAPDH) were quantified in separate tubes.

After Genomic DNA extraction and quantification, Genomic DNA sample concentrations were normalized to 3 ng µL^−1^. gDNA (1 µL) were amplified by PCR using the TB GreenPremix Ex Taq II Kit (RR820A, Takara) on a CFX96 Real‐Time PCR Detection System (Bio‐Rad). The Ct values of bacterial DNA per ng of total DNA were calculated.^[^ ^67^ ^]^

All primer sequence were as follows:

*VSIR*:forward: 5ʹ‐ACGCCGTATTCCCTGTATGTC‐3ʹ;
reverse: 5ʹ‐TTGTAGAAGGTCACATCGTGC‐3ʹ;
*CCL4L2*:forward: 5ʹ‐CCGCCTGCTGCTTTTCTTAC‐3ʹ;
reverse: 5ʹ‐TTGCTTGCCTACCACAGC‐3ʹ;
*MUC2*:forward: 5ʹ‐GAGCTCCAGACAGAGGGCAGA‐3ʹ;
reverse: 5ʹ‐GGTAGATGGTGTCATCCTTGATGG‐3ʹ;
*ZG16*:forward: 5ʹ‐CCGAACAGTAGAGGCCTTCC‐3ʹ;
reverse: 5ʹ‐CGCACCTGAAGACCTACGAT‐3ʹ;
*AGR2*:forward: 5ʹ‐AGAGCAGTTTGTCCTCCTCAA‐3ʹ; 
reverse: 5ʹ‐CAGGTTCGTAAGCATAGAGACG‐3ʹ;
*IL‐1β*:forward: 5ʹ‐CAGCTACGAATCTCCGACCACCAC‐3ʹ;
reverse: 5ʹ‐GCCTCGTTATCCCATGTGTCGAAG‐3ʹ;
*IL‐6*:forward: 5ʹ‐AGCAGCAAAGAGGCACTGGCAG‐3ʹ;
reverse: 5ʹ‐ATCTGCACAGCTCTGGCTTGTTCC‐3ʹ;
*GAPDH*:forward: 5ʹ‐GCACCGTCAAGGCTGAGAAC‐3ʹ;
reverse: 5ʹ‐TGGTGAAGACGCCAGTGGA‐3ʹ;
Universal bacteria: 8F: 5ʹ‐AGAGTTTGATYMTGGCTCAG‐3ʹ;
1510R: 5ʹ‐TACGGYTACCTTGTTACGACTT‐3ʹ.

### Western Blotting

Total proteins were extracted using radioimmunoprecipitation assay buffer and quantified using an enhanced bicinchoninic acid protein assay kit (P0010S, Beyotime). Equal amounts of protein were loaded, individualed to sodium dodecyl sulfate‐polyacrylamide gel electrophoresis, and then transferred to a polyvinylidene fluoride membrane. After blocking with 5% nonfat milk for 1 h at room temperature, the membrane was incubated with specific primary antibodies overnight in 4 °C, followed by incubation with a horseradish peroxidase secondary antibody (Jackson ImmunoResearch) for 1 h at room temperature. Proteins were detected using enhanced chemiluminescence (Perkin Elmer). The proteins were visualized using a chemiluminescence imaging system (Bio‐Rad).

The primary antibodies used for immunoblotting were rabbit anti‐VSIR (ab243891, Abcam;1:1000), rabbit anti‐MUC2 (A14659, Abclona; 1:1000), rabbit anti‐ZG16 (17397‐1‐AP, Proteintech; 1:1000), and rabbit anti‐AGR2 (12275‐1‐AP, Proteintech; 1:1000), mouse anti‐*β*‐actin (66009‐1‐Ig, Proteintech; 1:10000). The horseradish peroxidase‐conjugated secondary antibodies used were goat anti‐rabbit IgG H&L (HRP; ZB5301, Zsbio, Beijing, China; 1:5000) and goat anti‐mouse IgG H&L (HRP; ZB5305, Zsbio; 1:5000).

### Co‐Incubation of THP‐1 with Enterobacteriaceae and Enterocyte Stimulation

*E. faecalis, V. parvula*, and *S. Anginosus* strains were purchased from ATCC and reproduced in nutrient brain‐heart infusion broth (*E. faecalis* and *S. Anginosus*) for 24 h or the Columbia Blood Plate (*V. parvula*) for 48 h, and quantified by McFar Land. The bacterial density used was 1.0 × 10^9^ CFU mL^−1^. The  ratio of *E. faecalis, V. parvula*, and *S. Anginosus* was 1:2:1. Before co‐incubation with macrophages, THP‐1 cells were treated with phorbol 12‐myristate 13‐acetate (PMA; 100 ng mL^−1^) for 48 h to differentiate THP‐1 cells into macrophages. The stimulation ratio of the strains to macrophages was 1:100 (V:V). After 6 h of co‐incubation, the CM was collected, sterilized by filtration through a 0.22‐µm pore membrane, and then applied to enterocytes with/without VSIR antibody (20 µg mL^−1[^ ^68^ ^]^). The CM from macrophages alone was used as a control. After 18 h of co‐incubation, the cells and their CMs were analyzed.

### Transwell Analysis

THP‐1 cells were treated with PMA; 100 ng mL^−1^) for 48 h for differentiate THP‐1 into macrophage. Cells (1 × 10^5^) were plated in top chamber from 24‐well Transwell chamber with polyester membrane of 8‐µm pore size (3422, Corning). The bottom cha057mber was added with 0.5 mL medium containing *E. faecalis, V. parvula*, and *S. anginosus* mixed medium with/without CCL4L2 antibody (5 ng µL^−1^, which was used according our pre‐experiment). After 24 h, the non‐migrated cells on the top surface of the PET membrane were removed, and the migrated cells were fixed with 4% paraformaldehyde for 15 min, stained with 0.1% crystal violet for 10 min, and washed three times with PBS. Migrated cells were visualized under a microscope.

### Hematoxylin‐Eosin Staining

The hematoxylin‐eosin staining kit (E‐IR‐R117, Elabscience). The sections were soaked in xylene and a gradient of alcohol, and the prepared Weigert iron hematoxylin and eosin staining solution was used for sequential staining. Finally, the slices were sealed with neutral resin.

### Antibiotic Pre‐Clearance of Mouse Gut Microbiota

Mice aged 8–10 weeks were administered drinking water containing ampicillin (1 g L^−1^), metronidazole (1 g L^−1^), 1 g L^−1^ neomycin (1 g L^−1^), and vancomycin (0.5 g L^−1^) for 7 days. The mice were then administered an antibiotic cocktail via oral gavage for one week.

### Selected Bacterial Transplantation (SBT)

After gut microbiota clearance, mice were transplanted with microbiota cocktail containing *E. faecalis*, *S. anginosus* and *V. parvula* (1 × 10^9^ CFU/mouse/day), the dosage ratio of the bacterium was *E. faecalis*: *S. anginosus*: *V. parvula* = 1:1:2. The mice were administered 200 mL of the microbiota cocktail by gavage once a day for ten days, followed by once at 3‐day intervals for the following days.

### DSS‐Induced Acute Colitis

Mice were treated with 2.5% (w/v) dextran sulfate sodium (DSS, Millipore Corporation, Billerica, MA, USA) in drinking water after SBT for 7 days, along with an antibiotic cocktail (daily) or VSIR antibody (10 mg kg^−1^, once every 4‐day interval^[^ ^69^ ^]^) treatment. The changes in body weight and overall survival were recorded daily. On day 7, the mice were sacrificed, and the colon was separated for pathological analysis. All animal experiments were approved by the Animal Committee of the Guangdong Provincial People's Hospital.

### Statistical Analysis

Quantification of the covariation between factors from the metadata was performed by Mantel tests using the mantel.rtest function in the R package ape4. Co‐occurrence analysis was performed by calculating Spearman's rank correlations between continuous numerical factors and the incidence of comorbidities or selected paired genera. Significance of the differentiation of microbiota structure between groups were assessed by permutational multivariate analysis of variance (PERMANOVA) using R package “vegan.” Changes in microbial abundance between groups were statistically analyzed using the paired‐wise Wilcoxon test with Bonferroni‐Holm correction from the R stats package.

Statistical analyses and graphics were generated using GraphPad Prism A7 (GraphPad Software). Data were presented as mean ± standard error. The Shapiro–Wilk normality test was used to determine whether the data satisfied a normal distribution. Significant differences between groups were analyzed using one‐way analysis of variance (ANOVA) with multiple comparisons, followed by Dunnett's post hoc test for significance or two‐tailed Student's *t*‐test. The sample size for each statistical analysis was at less than 3. Statistical significance was set at *P* ≤ 0.05.

### Availability of Data and Materials

The processed gene expression and raw sequencing data that support the findings of this study have been deposited in the Genome Sequence Archive of the Beijing Institute of Genomics, Chinese Academy of Sciences, under accession numbers HRA003028 (https://ngdc.cncb.ac.cn/gsa‐human/browse/HRA003028) and CRA008088 (https://ngdc.cncb.ac.cn/gsa/browse/CRA008088). The shape data for the map of China is provided by the Resource and Environment Science and Data Center (https://www.resdc.cn/Datalist1.aspx?FieldTyepID = 20,0, accessed on 12 May 2022).

## Conflict of Interest

The authors declare no conflict of interest.

## Author Contributions

J.L., Y.Z., C.J., H.C., and Z.S. contributed equally to this work. M.Y., Y.G., S.G., and W.X. conceived and supervised the project. M.Y., Y.Z., H.C., J.W., X.Z., Y.L., Y.C., X.Z., H.Z., G.Z., Y.S., C.W., Y.H., H.Z., Y.W., Y.L., Y.W., X.J., H.S., Q.T., X.X., Y.X., L.Y., J.X., L.G., Z.L., X.W., R.M., and L.Z. provided patient care, clinical and histological assessments. W.X., L.L., Y.W., S.L., and N.M. performed the experiments. J., H., Z., and Z. performed the bioinformatics analysis. Z.S., X.Z., L.S., J.H., A.C., Y.W., X.P., and G.J. processed the clinical data. M.Y. wrote the manuscript with inputs from Z.Z., S.Z., S.G., and Z.S. All authors discussed and approved the manuscript.

## Supporting information

Supporting Information

Supporting Table 1

Supporting Table 2

Supporting Table 3

Supporting Table 4

Supporting Table 5

Supporting Table 6

Supporting Table 7

Supporting Table 8

Supporting Table 9

Supporting Table 10

Supporting Table 11

Supporting Table 12

Supporting Table 13

Supporting Table 14

Supporting Table 15

## Data Availability

The processed gene expression and raw sequencing data that support the findings of this study were deposited at the Genome Sequence Archive of the Beijing Institute of Genomics, Chinese Academy of Sciences under the accession number HRA003028 (https://ngdc.cncb.ac.cn/gsa‐human/browse/HRA003028) and CRA008088 (https://ngdc.cncb.ac.cn/gsa/browse/CRA008088).
