# Supplementary material for: Gut Dysbiosis Drives Inflammatory Bowel Disease Through the CCL4L2‐VSIR Axis in Glycogen Storage Disease
Source: Adv Sci (Weinh). 2024 Jun 18;11(30):2309471. doi: 10.1002/advs.202309471 (PMC11321658; doi:10.1002/advs.202309471)
Supplement: Supplementary file 1 — Supporting Information [file ADVS-11-2309471-s003.docx]

**Supplementary figures**

**
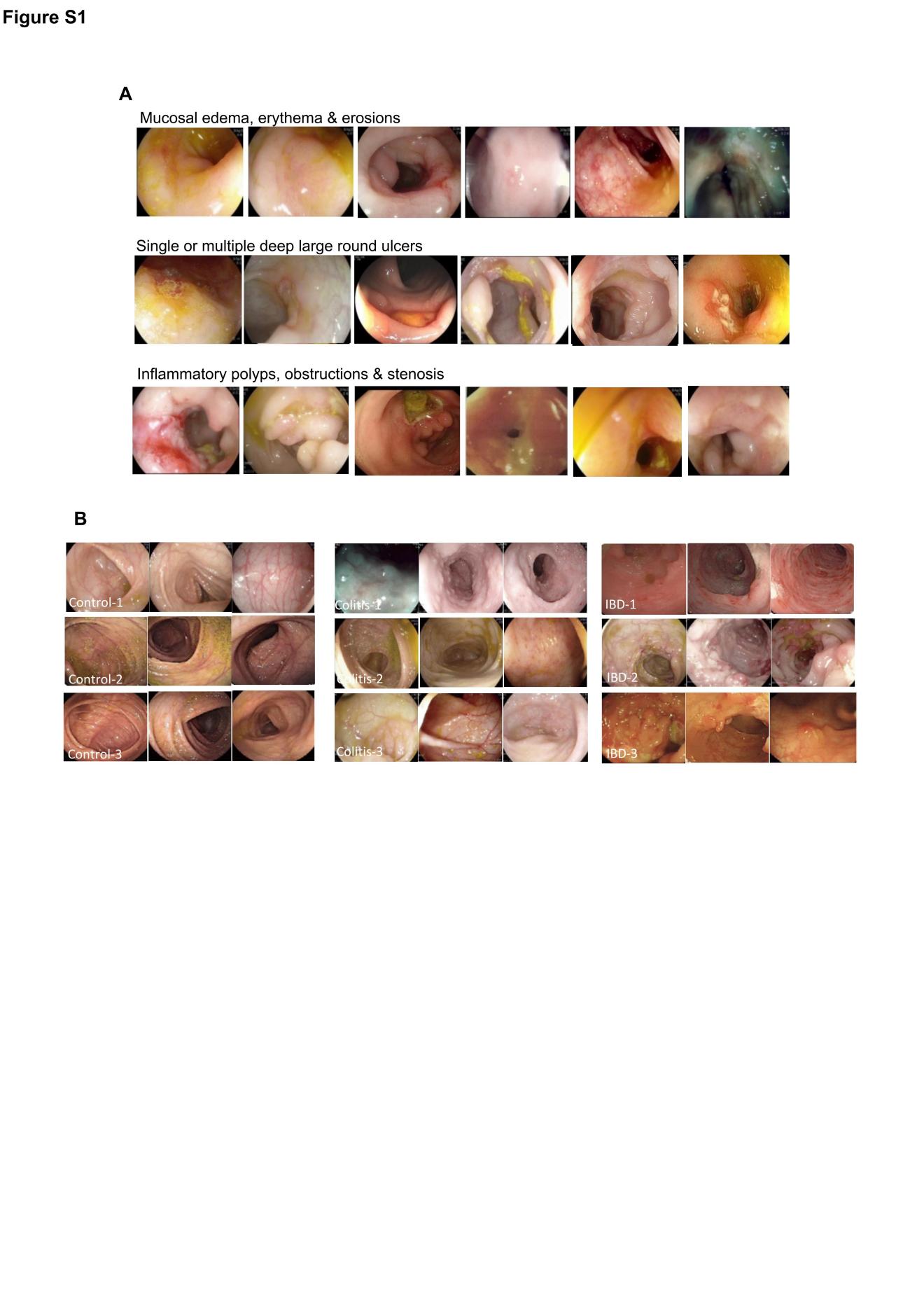
**

**Fig. S1** **Representative colonoscopy images for studied patients**

**A**. Endoscopic images characteristics as mucosal edema, erythema, erosions, single or multiple scattered deep round ulcers, inflammatory pseudo polyps, obstructions, and strictures in GSD-Ib.

**B**. Endoscopic images include nodularity, edema, erythema, ulcers, and inflammatory polyps for colitis and patients with IBD. Colonic biopsies from three groups for 5ʹ-end single-cell RNA transcriptome analysis. GSD, glycogen storage disease; IBD, inflammatory bowel disease

**
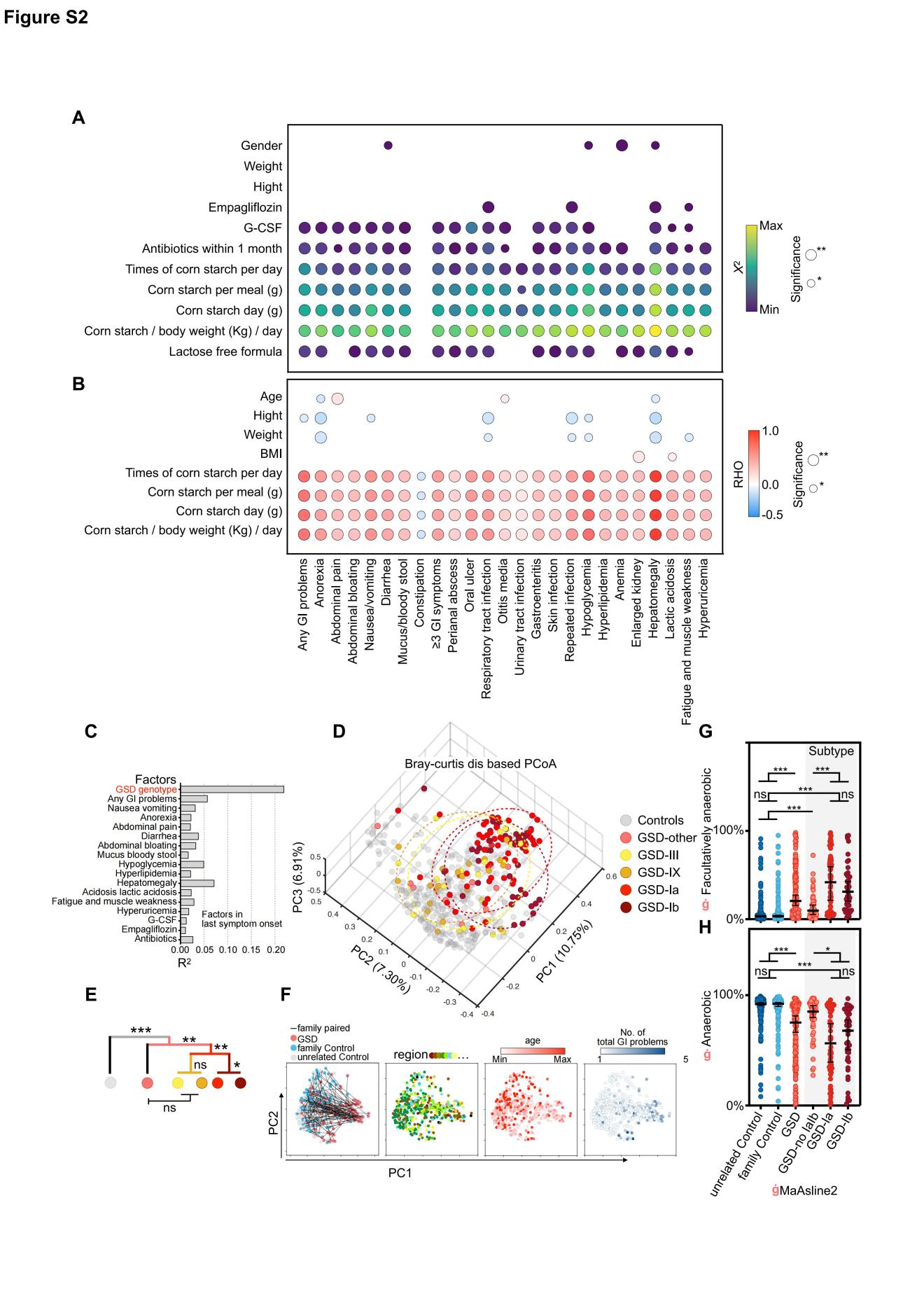
**

**Fig. S2 The genotype Ia and Ib dominate the gut microbiota shifts in patients with GSD**

**A**. Distributional correlation of other individual factors from metadata. Mantel test and FDR-corrected statistical significance.

**B**. Correlation of individual continuous-numerical factors and the incidence of comorbidities is obtained by spearman's rank correlation coefficient test. FDR-corrected statistical significance.

**C**. 17 factors in last symptoms onset from metadata showed significant impacts on gut microbiota and their amount effect size.

**D,E**. Detailed genotype grouped patient’s distribution on a three-dimensional principal coordinates analysis space (**D**) and significance (PERMANOVA respectively, **E**) of the variations inner the patients with GSD. The genotype of patients with sample size below 20 (0, IV and VI) are assembled as the GSD-other group.

**F**. Distribution of patients according the factors that with the most considerable impacts on their gut microbiota among the first and second coordinates (PC1 and PC2). Patients are colored according to preliminary group, region, age and total number of gastrointestinal problems. Lines in the left scatter diagram indicating the connected two patients are from one family.

**G,H**. Facultatively anaerobic (**G**) and anaerobic (**H**) level of gut microbiota assessed against Bugbase among each group. Median with 95% CI and paired-wise Wilcoxon test with Bonferroni–Holm Correction.

* *P* < 0.05, ** *P* < 0.01, *** *P* < 0.001 and **** *P* < 0.0001. GSD, glycogen storage disease; IBD, inflammatory bowel disease

**
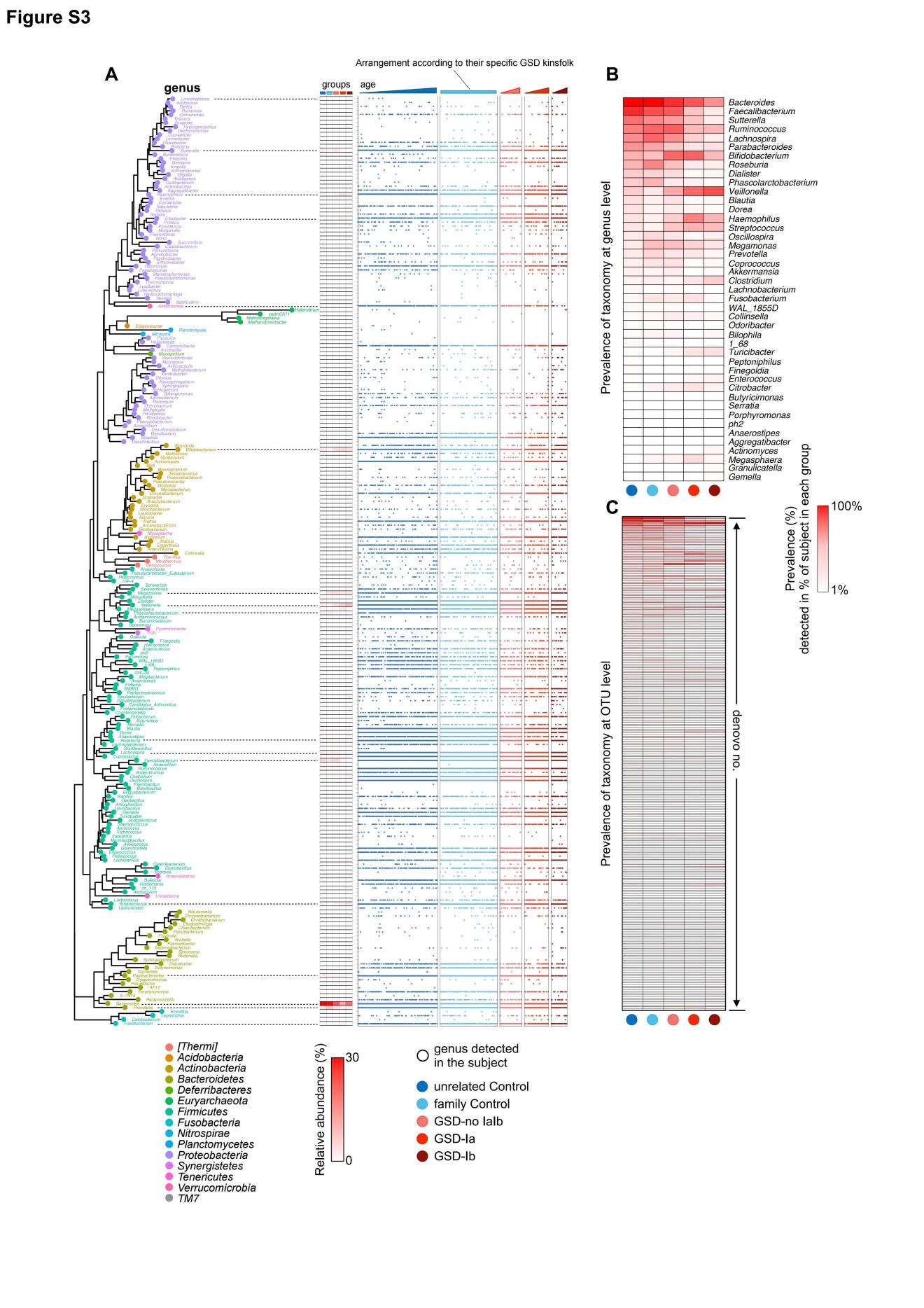
**

**Fig. S3 Phylogenetic relationship and prevalence of gut microbiota in each sample**

**A**. Neighbor-joining phylogenetic tree of all genera (n=233) assigned in our cohort. Heatmap shows the mean abundance of each genus in each group and the dots indicate the presence of the genera at any abundance in the specific sample.

**B,C**. The prevalence of each genus (**B**) and OTU level taxa (**C**) among groups. Only genera or OTUs with detection rate above 1% are exhibited. OTU, operating taxonomic unit

**
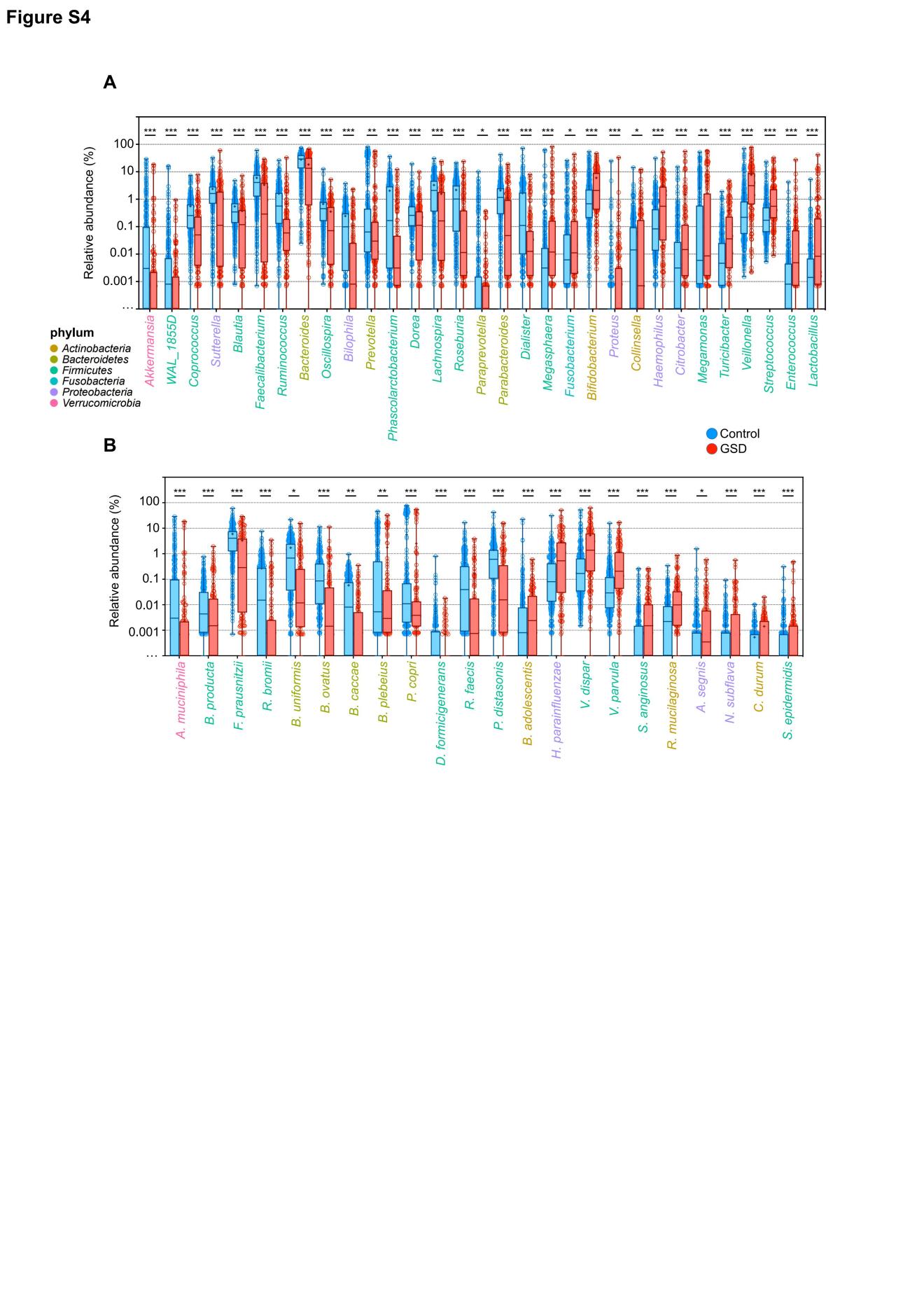
**

**Fig. S4 Microbiota signatures with significant abundance change between Control and GSD**

Box-whisker plots illustrating the abundance shifts of gut microbiota at the genus (**A**) and species (**B**) levels in the control and GSD groups. + indicates mean abundance. Paired-wise Wilcoxon test with Bonferroni–Holm Correction. * *P* < 0.05, ** *P* < 0.01, *** *P* < 0.001. GSD, glycogen storage disease

**
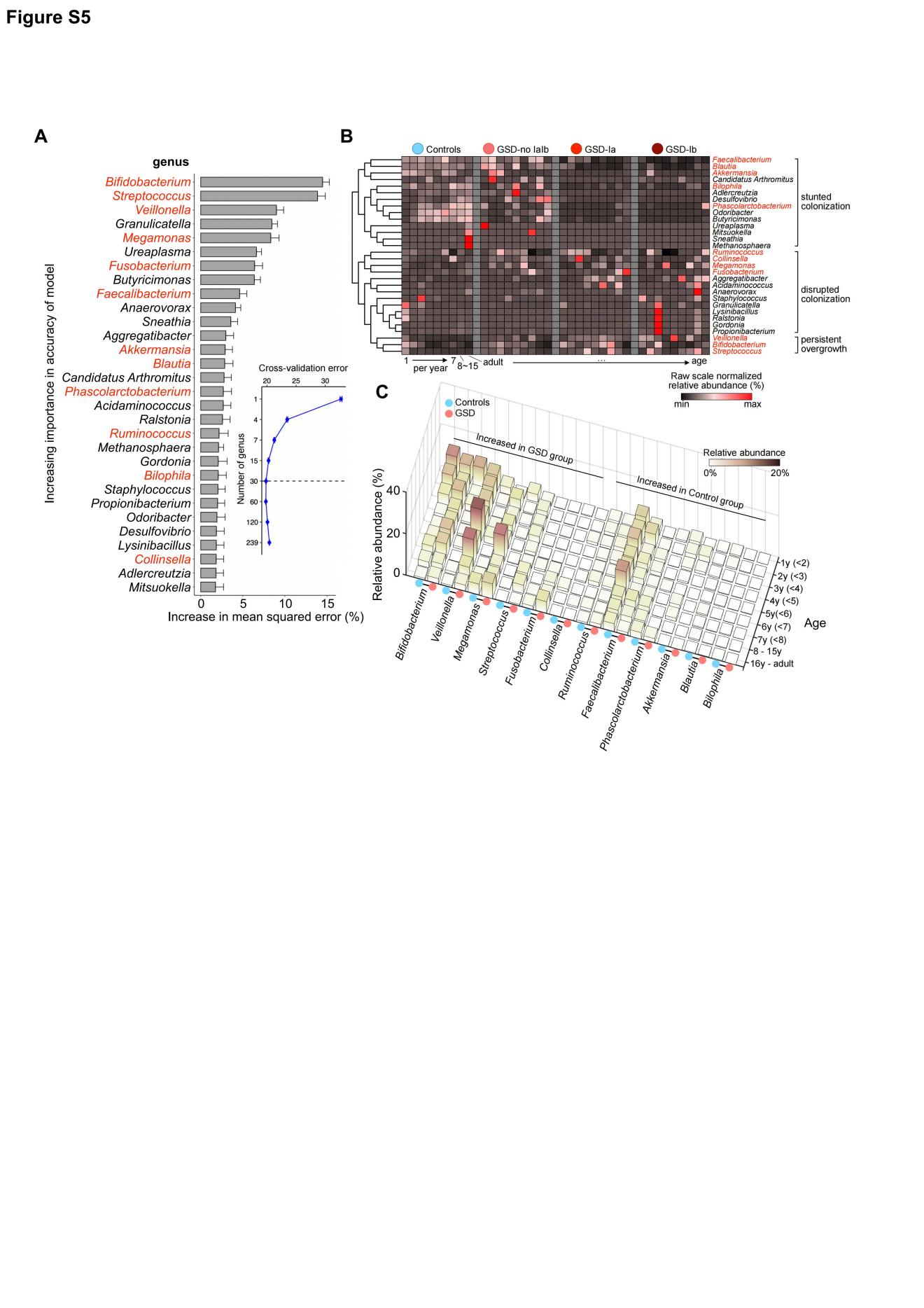
**

**Fig. S5 Disrupted development of age-discriminatory taxa in GSD**

**A**. Thirty genera were identified as age-discriminatory bacterial taxa via random forest regression and ranked in descending order of their importance to the accuracy of the model. Importance was determined based on the percentage increase in mean-squared error of microbiota age prediction when the relative abundance values of each taxon were randomly permuted. Importance was determined based on the percentage increase in mean-squared error of microbiota age prediction when the relative abundance values of each taxon were randomly permuted (mean importance ± s.d. and n=100 replicates). The insert shows five times 10-fold cross-validation error as a function of the number of input bacterial genus (blue line).

**B**. Mean relative abundance of the 30 age-discriminatory genera plotted against the chronologic age in healthy controls, respectively. Clusters performed according to the abundance distributional metric of each genus against age.

**C**. Grouped average relative abundance of the 12 age-discriminatory genera with both significant inter group abundance changes in Control and GSD across age. GSD, glycogen storage disease

**
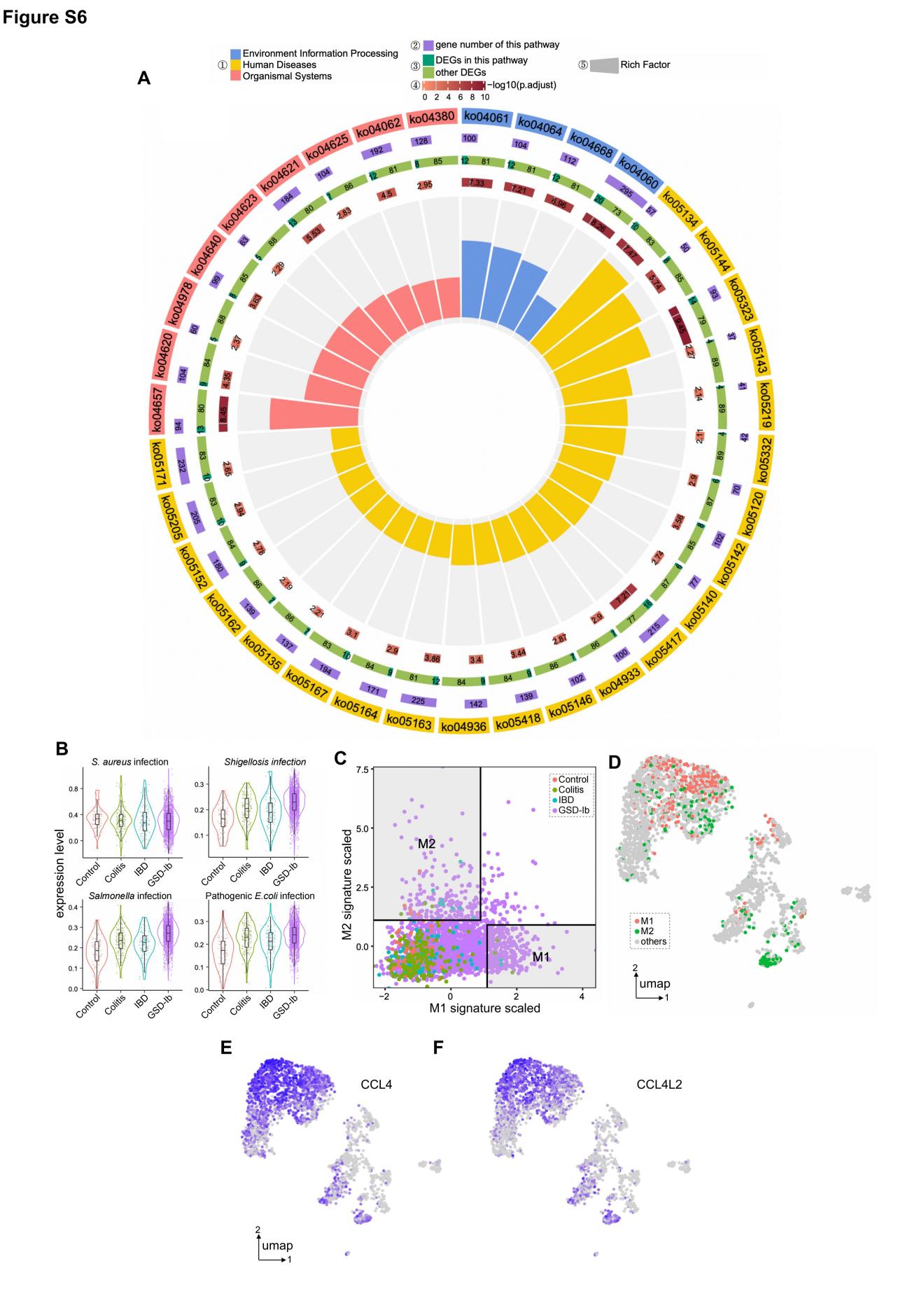
**

**Fig. S6 Pathogenic infection associated polarized characteristics of macrophages**

**A**. KEGG enrichment analysis of the upregulated genes in GSD-Ib versus other groups macrophages. The first lap indicates KEGG pathway entry ID and annotation. The second lap indicates the gene number of pathway. The third lap indicates the number of DEGs belong to pathway (deep green) and other DEGs (light green). The fourth lap indicates “−log10 (*P*.adjust)” and grey line indicates adjusted *P* value 0.01. The fifth lap indicates enrichment factor of each KEGG pathway.

**B**. Violin plots and box plots showing the expression of selected signatures in different groups.

**C**. Visualization of M1 and M2 macrophage polarization states defined by scaled M1 and M2 signature scores.

**D**. UMAP visualization of macrophages colored by macrophage polarization states.

**E,F**. Feature plot showing the expression of CCL4 (**E**) or CCL4L2 (**F**). GSD, glycogen storage disease

**
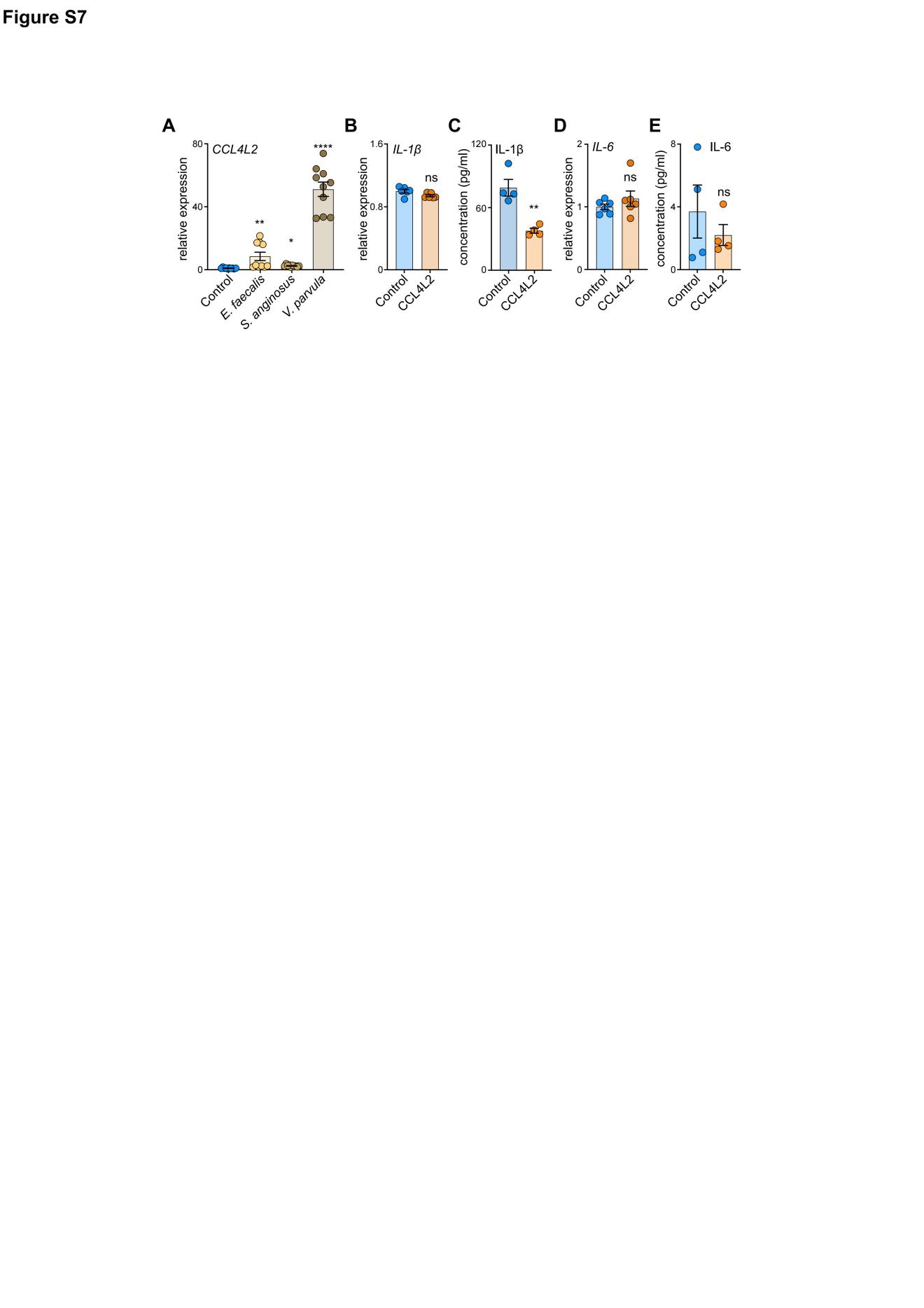
**

**Fig. S7 Intestinal bacteria-derived CCL4L2 to triggers inflammatory factors in macrophages**

1. Intestinal microbiota promotes the expression of CCL4L2 in THP-1-derived macrophages (n=10). *P* values were calculated using unpaired *t* test.

**B,C**. The Secreted IL-1β protein level (n=4) and mRNA level (n=6) of IL-1β were determined in THP-1-derived macrophages treated with CCL4L2. *P* values were calculated using unpaired *t* test.

**D,E**. Secreted IL-6 protein (n=4) and mRNA (n=6) of IL-6 were determined in THP-1-derived macrophages treated with CCL4L2. *P* values were calculated using unpaired *t* test.

Data are presented as mean ± SEM; **P* < 0.05, ***P* < 0.01, *****P* < 0.0001, ns: not significant.

**
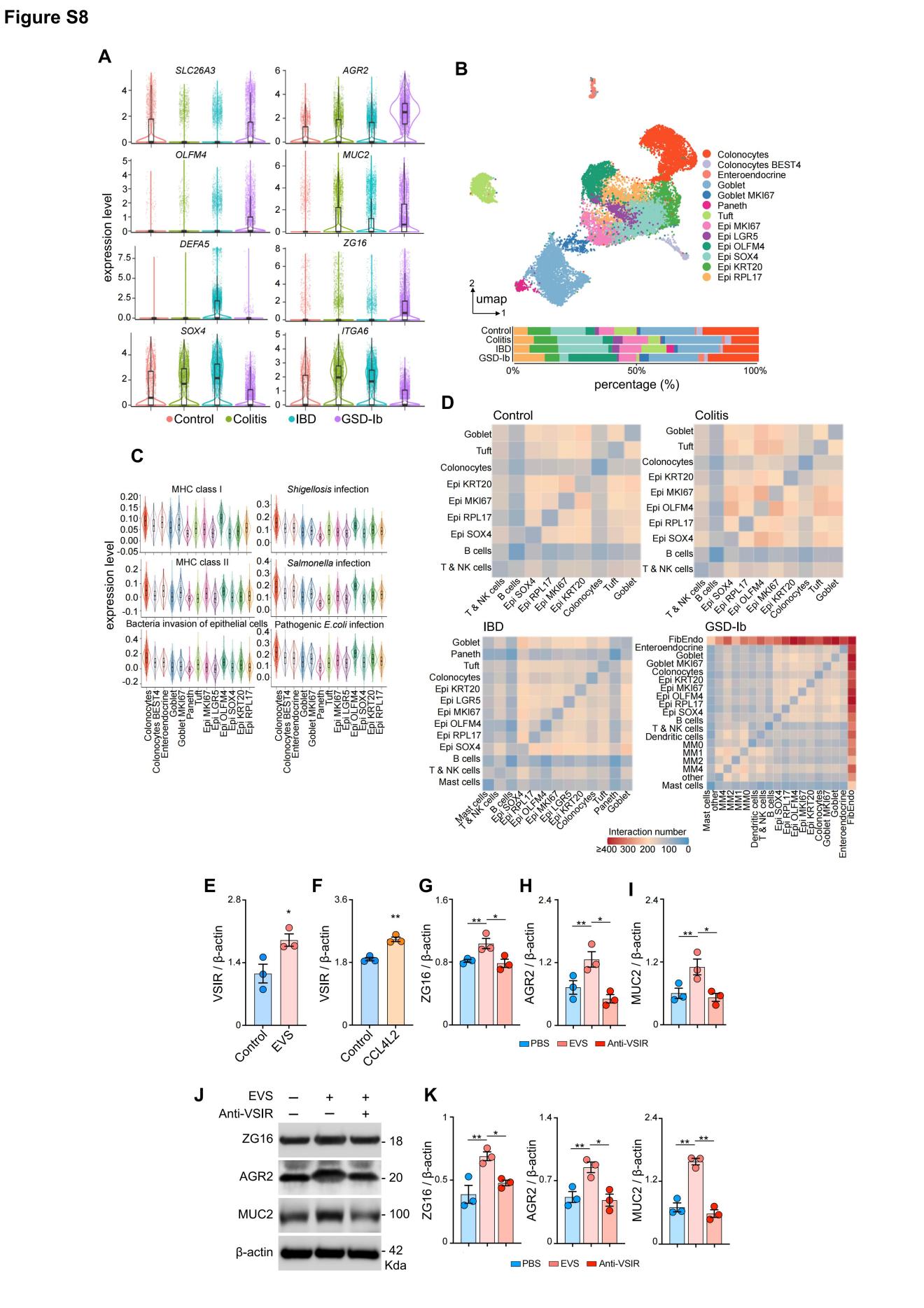
**

**Fig. S8 Infection-related pathways involved interaction between macrophage and non-immune cells**

**A**. Violin plots and box plots showing the expression of selected differentially expressed genes between different groups.

**B**. UMAP plot displaying color-coded epithelial subclusters. Bar chart showing the proportion of different subclusters in different groups.

**C**. Violin plots and box plots showing the expression of selected signatures in different subclusters.

**D**. Heatmap showing the total number of interactions between cell types. Considering the possibility of cell-cell interaction, cell types accounting for less than 5‰ in each group were removed.

**E–I.** Bar charts showing the quantification of the western blotting analysis (n=3). *P* values were calculated by the unpaired *t* test (E, F) or the One-ANOVA (G–I).

**J,K.** Western blotting analysis and quantification showing the enhanced ZG16, AGR2, and MUC2 expression in CaCO2 cells treated with CM from indicated group for 24 h*,* respectively (n=3). *P* values were calculated by the One-ANOVA.

Data are represented as mean ± SEM. **P* < 0.05, ***P* < 0.01, *****P* < 0.0001. CM, conditional medium.

**
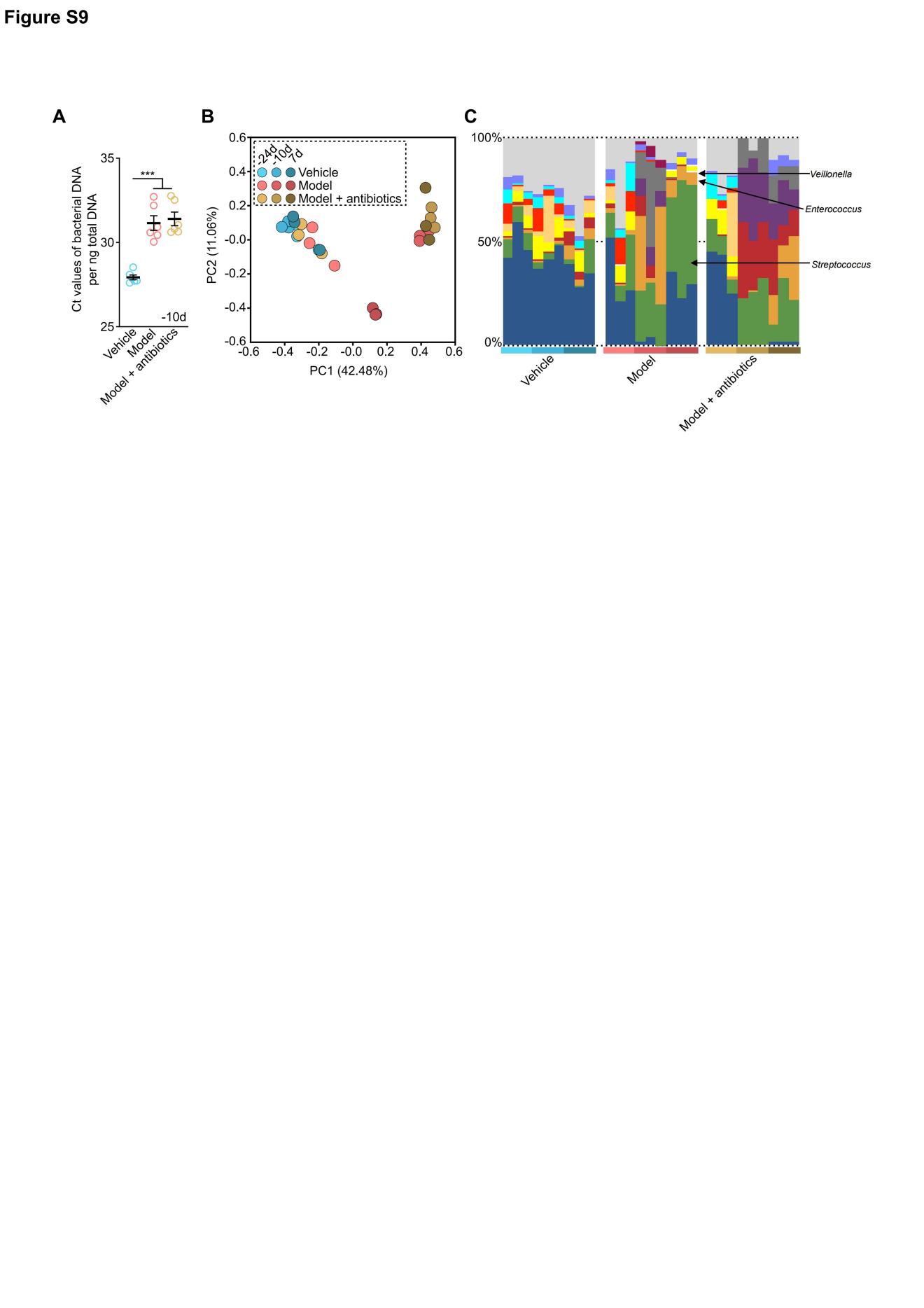
**

**Fig. S9 Murine gut microbial change during model constructions**

**A**. Ct values of the universal bacterial DNA per 1 ng of total DNA in stools. All DNA samples are duplicated detected.

**B**. The PCoA space showing the gut microbial composition shift between groups. Plots representing the specific mice are colored according to their groups.

**C**. Relative abundance genus-level gut microbiota composition in each mice and, locations of *Veillonella*, *Enterococcus* and *Streptococcus* are given.

Data are represented as mean ± SEM; *P* values were calculated by the unpaired *t* test. ****P* < 0.001.
